# A Physics-Informed Multi-Task Soft Sensor for Uncertainty-Aware Slag Tin Content Prediction in Top-Blown Tin Smelting

**DOI:** 10.3390/s26165157

**Published:** 2026-08-14

**Authors:** Zhaojun Ma, Jubo Peng, Xiaojun Zhou, Kui Xie, Zhang Zhang

**Affiliations:** 1 School of Resources and Environment, Yunnan University, Kunming 650500, China; mazhaojun@ytc.cn; 2 Yunnan Tin Group (Holding) Co., Ltd., No. 471 Minhang Road, Guandu District, Kunming 650200, China; zhangzhang@ytc.cn; 3 School of Automation, Central South University, Changsha 410083, China; 244612215@csu.edu.cn

**Keywords:** soft sensor, slag tin content, top-blown tin smelting, sparse labels, physics-informed learning, multi-task learning, uncertainty quantification, industrial process monitoring

## Abstract

Slag tin content is a key indicator of reduction efficiency and metal recovery in top-blown tin smelting, but delayed and sparse laboratory assays hinder online monitoring. This study proposes a physics-informed multi-task soft sensor (PI-MTSS) combining a shared temporal convolutional encoder, self-supervised future state reconstruction, a soft monotonicity constraint based on a simplified CO–temperature reduction-potential indicator, and Monte Carlo dropout for epistemic uncertainty estimation. The model was evaluated on a continuous 30-day dataset comprising 43,200 one-minute process observations and 240 laboratory assays, including 40 chronologically held-out test labels. Across five random seeds, PI-MTSS achieved an RMSE of 0.198, an MAE of 0.145, and an $R^{2}$ of 0.889. Across three rolling-origin evaluations, it obtained a mean RMSE of 0.199 and maintained the lowest RMSE among the evaluated methods. The complete model reduced the physical violation rate to 3.5%. At 95% nominal coverage, its epistemic intervals achieved a PICP of 96.5% and an NMPIW of 0.245; the ACE across six nominal levels was 0.021, and uncertainty correlated positively with absolute error ($\rho_{s}=0.691$). These results support the potential of PI-MTSS for minute-scale monitoring and operator decision support under sparse labels, although validation remains limited to one month from a single furnace.

## 1. Introduction

Slag tin content is one of the key quality indicators for evaluating reduction efficiency, metal recovery, and operating stability in top-blown tin smelting. A high residual tin content in slag usually indicates insufficient reduction and increased metal loss, whereas inappropriate adjustment of the reducing atmosphere may also lead to unstable furnace conditions and unnecessary energy consumption. Therefore, timely and reliable monitoring of slag tin content is important for process assessment, operation optimization, and intelligent control in pyrometallurgical production [1,2,3]. In top-blown smelting furnaces, process variables such as rising flue temperature and off-gas composition are closely related to the furnace state and are widely used by operators to evaluate the reduction condition and production stability [1,2,4].

However, slag tin content is difficult to measure online using conventional physical sensors. In actual industrial plants, high-frequency process variables, including off-gas components, furnace temperature, oxygen-enriched air flow, and other distributed control system (DCS) signals, can be continuously recorded at short sampling intervals. By contrast, slag tin content is usually obtained through manual sampling and offline laboratory assays, which introduces both measurement delay and label sparsity [5,6]. In the industrial dataset considered in this study, process variables were recorded every minute, whereas slag tin labels were available approximately every three hours, yielding 43,200 high-frequency process samples but only 240 laboratory labels in one month. This multi-rate measurement structure leads to a typical soft sensing problem: the target quality variable is sparse and delayed, while the auxiliary process measurements are abundant and continuously available.

Data-driven soft sensors provide a practical solution for estimating difficult-to-measure quality variables from easily accessible process measurements [7,8,9]. In recent years, machine learning and deep learning models have been widely applied to industrial process monitoring and quality prediction. Sequential models, including long short-term memory (LSTM) networks, temporal convolutional networks (TCNs), and spatiotemporal deep learning models, have shown strong capability in capturing nonlinear temporal dependencies in complex industrial processes [10,11,12,13]. These methods have improved the prediction accuracy of process soft sensors in many chemical and metallurgical applications. Nevertheless, most existing supervised soft sensing models implicitly assume that sufficient labeled samples are available. This assumption is often not valid in top-blown tin smelting, where laboratory assays are expensive, delayed, and sparsely sampled. Training a purely supervised model with only a limited number of slag labels may result in overfitting and weak generalization, while the large amount of unlabeled high-frequency process data remains insufficiently exploited.

Recent advances in adaptive soft sensing and dynamic representation learning have improved industrial quality prediction under complex operating conditions. For example, self-modified dynamic domain adaptation addresses distribution shifts through pseudolabel refinement and feature alignment [14], while GraphSiamese captures spatial–temporal dependencies among process variables using graph-based slow feature learning [15]. However, existing methods mainly focus on cross-domain adaptation or dynamic feature representation, and generally do not jointly address three key practical challenges in industrial smelting: sparse laboratory labels, insufficient utilization of unlabeled high-frequency process data, and the lack of physical consistency and uncertainty information in model predictions.

Semi-supervised learning has been investigated to alleviate the dependence on labeled data in industrial soft sensing [9,16,17]. Existing methods mainly include pseudolabeling, consistency regularization, semi-supervised autoencoders, and multi-task learning frameworks [18,19,20]. Although these methods are effective in some industrial applications, pseudolabeling may introduce error propagation when applied to highly dynamic metallurgical processes, as inaccurate pseudolabels generated in the early training stage can bias the subsequent learning process. Moreover, many semi-supervised soft sensors focus mainly on improving fitting accuracy and do not explicitly model the temporal evolution patterns embedded in unlabeled process trajectories. For top-blown smelting, high-frequency off-gas and temperature sequences contain important information about furnace dynamics. Therefore, a self-supervised auxiliary task that learns dynamic representations from unlabeled process data is more suitable for sparse-label slag tin prediction than directly assigning pseudolabels to the quality variable [19,21,22].

Another limitation of conventional soft sensors is their deterministic output form. Standard neural network-based regression models usually provide only a point estimate, even when the input condition is far from the training distribution. In real industrial operation, furnace states may shift due to changes in feed composition, lance conditions, oxygen supply, coal injection, or abnormal fluctuations. Under these out-of-distribution (OOD) conditions, a deterministic soft sensor may still produce an apparently precise prediction without indicating its reliability, leading to overconfident decisions in data-sparse regions [23,24]. Bayesian neural networks and ensemble-based methods can be used to estimate predictive uncertainty, but often increase computational complexity or require additional model training. Monte Carlo (MC) dropout offers a lightweight alternative by approximating Bayesian inference through stochastic forward passes, enabling the model to provide both predictive means and confidence intervals [25,26]. For industrial soft sensors, uncertainty quantification is valuable not only for evaluating prediction reliability but also for providing risk-aware warnings during unfamiliar operating conditions [27,28].

In addition to data sparsity and uncertainty, physical plausibility is also essential for soft sensors used in metallurgical processes. Purely data-driven models may learn spurious correlations from sparse and noisy data, especially near the boundary of the operating space. As a result, their predictions may violate basic metallurgical knowledge. In top-blown tin smelting, the residual tin content in slag is closely related to the carbothermal reduction process. A stronger reducing atmosphere, reflected by higher CO-related reduction-condition proxy index and suitable furnace temperature, should generally promote the reduction of tin oxide and decrease the residual tin content in slag. Physics-informed machine learning provides a way to incorporate prior knowledge into data-driven models [29,30]. Physics-informed neural networks usually embed explicit differential equations into the loss function [31,32]; however, directly formulating accurate and tractable governing equations for gas–liquid–solid multiphase reactions in an industrial top-blown furnace is difficult. A more practical strategy is to introduce soft physical constraints derived from dominant metallurgical mechanisms. Such constraints can regularize model training and reduce physically inconsistent predictions without requiring a complete first-principles furnace model [33,34,35].

To address the above challenges, this study proposes a physics-informed multi-task soft sensor (PI-MTSS) for uncertainty-aware slag tin content prediction in top-blown tin smelting. The proposed method uses a TCN as the shared temporal encoder to extract dynamic features from sliding window process measurements. To exploit unlabeled high-frequency data, a self-supervised future state reconstruction branch is introduced as an auxiliary task, while the primary branch predicts slag tin content using sparse laboratory labels. To improve the physical reliability of the model, a monotonicity constraint based on the carbothermal reduction mechanism is incorporated into the training objective. MC dropout is further used to quantify predictive uncertainty and construct confidence intervals for risk-aware process monitoring.

The main contributions of this work are summarized as follows:

- A PI-MTSS is developed for online slag tin content prediction in top-blown tin smelting, where the target quality variable is delayed and sparsely labeled while high-frequency process measurements are continuously available.
- A self-supervised future state reconstruction task is introduced to exploit unlabeled process trajectories and improve temporal representation learning under sparse laboratory labels.
- A reduction mechanism-based monotonicity constraint is incorporated into the optimization objective to regularize the model with metallurgical prior knowledge and suppress physically inconsistent predictions.
- An uncertainty quantification strategy based on MC dropout is integrated into the soft sensor to provide prediction intervals and support risk-aware monitoring under unfamiliar furnace conditions.

The remainder of this paper is organized as follows: Section 2 describes the top-blown tin smelting process, industrial dataset, problem formulation, and proposed PI-MTSS method: Section 3 presents the experimental results and comparisons with baseline soft-sensing models: Section 4 discusses the effects of the self-supervised branch, physical constraint, and uncertainty quantification; finally, Section 5 concludes the paper and outlines future work.

## 2. Materials and Methods

### 2.1. Industrial Process and Sensor Measurements

**Figure 1:**
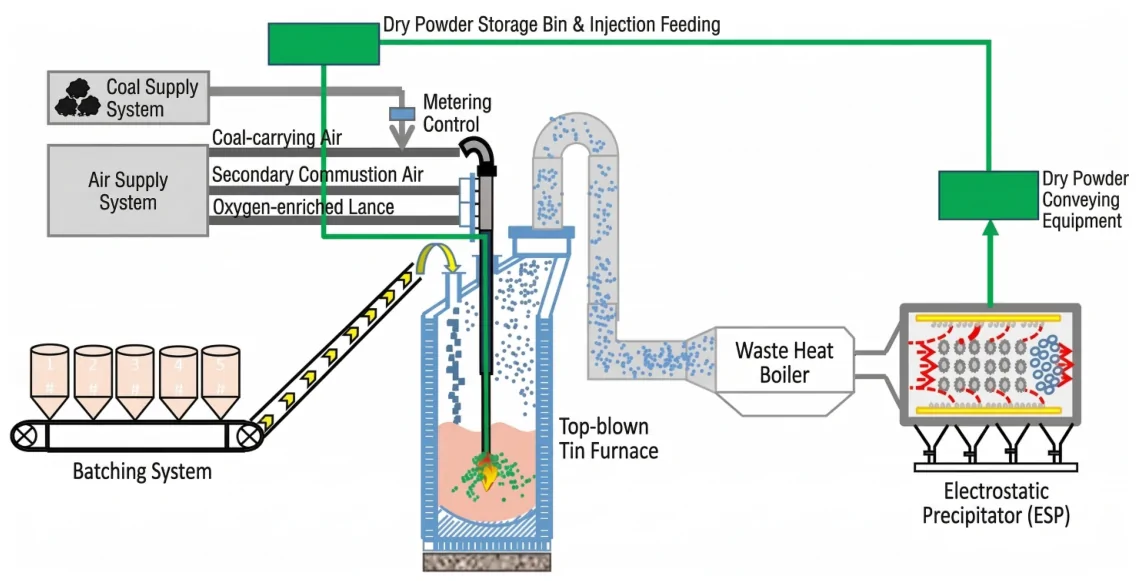
Schematic diagram of the top-blown tin smelting process. The main process units include the batching system, coal supply system, air supply system, oxygen-enriched lance, top-blown furnace, waste heat boiler, electrostatic precipitator, and dry powder recycling system.

The investigated process is a top-blown tin smelting furnace in an industrial pyrometallurgical production line. As shown in Figure 1, the furnace is a vertical reactor in which gas, molten slag, and molten metal interact under conditions of high temperature and strong turbulence. Tin concentrate, fluxes, and reducing agents are charged into the furnace, while oxygen-enriched air and fuel are injected into the molten bath through a submerged lance. This operation promotes rapid heat transfer, mass transfer, and reduction reactions, and finally produces crude tin, slag, and off-gas [1,2,3].

The smelting process is mainly governed by carbothermal reduction. The reducing agent first generates carbon monoxide through incomplete combustion and the Boudouard reaction:

(1) $$C+{\mathrm{CO}}_{2}\to 2\mathrm{CO}.$$

Then, tin oxide in the slag phase is reduced by carbon monoxide:

(2) $${\mathrm{SnO}}_{2}+2\mathrm{CO}⇌\mathrm{Sn}+2{\mathrm{CO}}_{2}.$$

According to this mechanism, the residual tin content in slag is closely related to the furnace temperature and the reducing atmosphere, which can be partially reflected by off-gas composition and thermal measurements. Therefore, high-frequency process measurements such as CO concentration, oxygen-related signals, SO_2_ concentration, furnace temperature, and other DCS variables provide useful information for estimating slag tin content.

The temperature signal and the concentrations of CO, O_2_, and SO_2_ were acquired by the installed industrial sensors and recorded by the distributed control system of the operating plant. Specific manufacturer and model information for the plant instrumentation cannot be disclosed under the industrial confidentiality agreement. Slag tin content was determined through the plant’s routine offline laboratory assay procedure.

However, slag tin content cannot be directly measured online in routine industrial operation. It is obtained through manual sampling and offline laboratory assays, which introduces measurement delay and sparse labels [5,6]. In the dataset used in this study, process variables were recorded every minute, whereas slag tin content was assayed approximately every three hours. As a result, the one-month dataset contained 43,200 high-frequency process samples but only 240 laboratory labels. This significant difference in sampling frequency forms a typical multi-rate soft sensing problem.

### 2.2. Data Preprocessing and Window Construction

Let $x_{t}\in R^{F}$ denote the vector of process variables sampled at time step *t*, where *F* is the number of measured process features. Since different variables have different physical units and numerical scales, all process variables are normalized using the Z-score method:

(3) $${\tilde{x}}_{t,f}=\frac{x_{t,f}-\mu_{f}}{s_{f}}$$

where $x_{t,f}$ is the original value of the *f*-th variable at time step *t*, while $\mu_{f}$ and $s_{f}$ are the mean and standard deviation of the *f*-th variable calculated from the training set.

To capture the dynamic evolution of the furnace state, a sliding window strategy is used to construct the model input. For the current time step *t*, the input sequence is defined as

(4) $$X_{t}=\left[{\tilde{x}}_{t-T+1},{\tilde{x}}_{t-T+2},\ldots ,{\tilde{x}}_{t}\right]\in R^{T\times F},$$

where *T* is the window length. In this study, *T* is set to 6 h, corresponding to 360 one-minute process samples. This window length is used to describe the historical evolution of the furnace before each slag assay.

The target variable is the slag tin content $y_{t}\in R$. Because slag labels are only available at sparse assay times, a binary label mask $m_{t}$ is introduced:

(5) $$m_{t}=\left\{\begin{array}{cc} 1, & \mathrm{if}\;\mathrm{the}\;\mathrm{laboratory}\;\mathrm{label}\;y_{t}\;\mathrm{is}\;\mathrm{available},\\ 0, & \mathrm{otherwise}. \end{array}\right.$$

The dataset can then be divided into a labeled subset and an unlabeled subset:

(6) $$D_{L}=\left\{\left(X_{i},y_{i}\right)|m_{i}=1\right\},\;D_{U}=\left\{X_{j}|m_{j}=0\right\}.$$

The objective of the soft sensor is to learn a mapping function

(7) $$f_{\Theta }:X_{t}\to {\hat{y}}_{t},$$

where ${\hat{y}}_{t}$ is the predicted slag tin content and Θ denotes all trainable parameters. The model should minimize the prediction error on $D_{L}$ while using the temporal structure of $D_{U}$ to improve representation learning and physical consistency.

### 2.3. Overall Architecture of PI-MTSS

**Figure 2:**
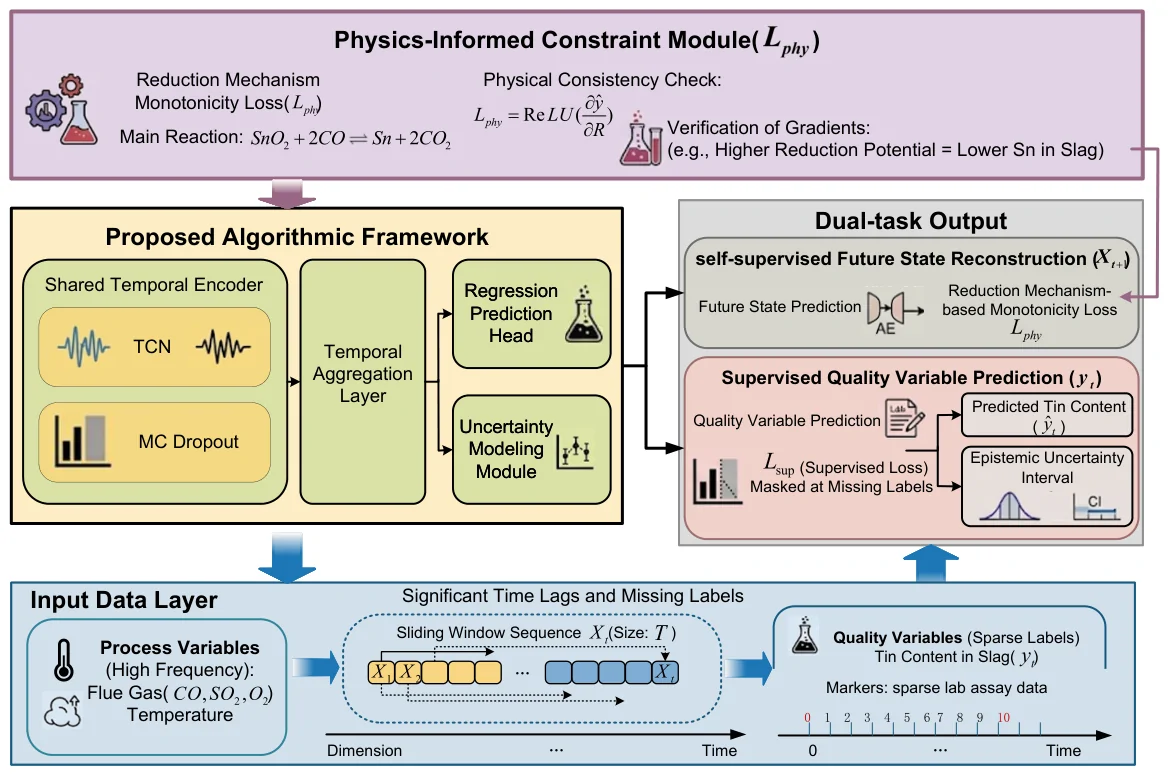
Overall architecture of the proposed PI-MTSS. The framework integrates high-frequency process measurements and sparse slag assay labels through a sliding window input layer. A shared temporal encoder extracts dynamic features, while the dual-task output module performs self-supervised future state reconstruction and supervised slag tin content prediction. The physics-informed constraint module regularizes the model using the reduction mechanism-based monotonicity loss, and MC Dropout provides uncertainty estimates for risk-aware monitoring.

To address label sparsity, prediction uncertainty, and physical inconsistency, we propose PI-MTSS. The overall architecture is illustrated in Figure 2. PI-MTSS consists of four main components: an input windowing module, a shared temporal encoder, a dual-task output module, and a physics-informed constraint module.

High-frequency process measurements and sparse slag labels are organized into unified input samples through the sliding window mechanism, allowing the model to describe furnace evolution before each assay time. Each input sequence $X_{t}$ is processed by the shared temporal encoder to extract latent dynamic features. The learned representation is then shared by a self-supervised reconstruction branch and a supervised regression branch. The reconstruction branch exploits unlabeled high-frequency data by learning to predict future process states, whereas the regression branch estimates slag tin content at the available laboratory assay times. During inference, MC dropout remains active to generate stochastic predictions, from which the predictive mean and uncertainty are calculated. Meanwhile, the physics-informed monotonicity constraint regularizes the model according to the expected inverse relationship between reduction-condition proxy index and residual slag tin content, thereby improving both prediction reliability and metallurgical plausibility.

### 2.4. Shared Temporal Encoder

A TCN is adopted as the shared temporal encoder because it can efficiently model long-range temporal dependencies through causal and dilated convolutions. For an input window $X_{t}$, the encoder maps the historical process sequence into a latent feature representation:

(8) $$z_{t}=E_{\theta }\left(X_{t}\right)$$

where $E_{\theta }\left(\cdot \right)$ denotes the TCN encoder and $z_{t}\in R^{D}$ is the latent feature vector.

The causal convolution ensures that the representation at time *t* only depends on current and historical process measurements. The dilated convolution expands the receptive field without excessively increasing the network depth. For a one-dimensional sequence, the dilated convolution can be expressed as

(9) $$h_{t}^{\left(l\right)}=\sum_{k=0}^{K-1}w_{k}^{\left(l\right)}h_{t-d_{l}k}^{\left(l-1\right)},$$

where $h_{t}^{\left(l\right)}$ is the hidden state of the *l*-th layer, *K* is the convolution kernel size, $d_{l}$ is the dilation factor, and $w_{k}^{\left(l\right)}$ is the convolution weight.

To support uncertainty estimation, dropout layers are embedded in the temporal encoder and kept active during stochastic inference. Therefore, the encoder can produce stochastic latent representations under different dropout masks:

(10) $$z_{t}^{\left(n\right)}=E_{\theta }\left(X_{t};\omega_{n}\right)$$

where $\omega_{n}$ denotes the dropout mask in the *n*-th stochastic forward pass.

### 2.5. Dual-Task Output Module

#### 2.5.1. Self-Supervised Future State Reconstruction

The first output branch is designed to reconstruct the future process state from the latent representation. This task does not require slag tin labels, so the training can use both labeled and unlabeled process data. Given $z_{t}$, a decoder network $G_{\phi }\left(\cdot \right)$ predicts the next-step process variable vector:

(11) $${\hat{x}}_{t+1}=G_{\phi }\left(z_{t}\right).$$

The reconstruction loss is defined as the mean squared error between the predicted future state and the observed future state:

(12) $$L_{\mathrm{rec}}=\frac{1}{M}\sum_{i=1}^{M}{∥{\tilde{x}}_{i+1}-{\hat{x}}_{i+1}∥}_{2}^{2},$$

where *M* is the number of samples in a mini-batch. By minimizing $L_{\mathrm{rec}}$, the shared encoder is encouraged to learn the temporal evolution patterns of the furnace from abundant high-frequency process data.

#### 2.5.2. Sparse Regression and Uncertainty Quantification

The second output branch predicts the slag tin content. In the complete PI-MTSS model, the latent temporal representation is augmented with the standardized reduction-condition proxy index defined in Section 2.6. Therefore, the regression output is calculated as

(13) $${\hat{y}}_{t}=H_{\psi }\left(\left[z_{t},{\tilde{R}}_{t}\right]\right),$$

where $H_{\psi }\left(\cdot \right)$ denotes the regression head, $z_{t}$ is the latent representation extracted by the shared TCN encoder, ${\tilde{R}}_{t}$ is the dimensionless standardized reduction-condition proxy index, and [·,·] denotes feature concatenation. Because slag labels are only available at sparse assay times, the supervised loss is calculated only when $m_{t}=1$. For a mini-batch, the masked supervised regression loss is defined as

(14) $$L_{\sup}=\frac{\sum_{i=1}^{M}m_{i}{\left(y_{i}-{\hat{y}}_{i}\right)}^{2}}{\sum_{i=1}^{M}m_{i}+\varepsilon },$$

where ε is a small constant used to avoid division by zero when no labeled sample appears in the mini-batch.

During inference, MC Dropout is used to estimate predictive uncertainty. For each input window $X_{t}$, the model performs *N* stochastic forward passes and obtains a set of predictions

(15) $$\left\{{\hat{y}}_{t}^{\left(1\right)},{\hat{y}}_{t}^{\left(2\right)},\ldots ,{\hat{y}}_{t}^{\left(N\right)}\right\}.$$

The predictive mean is calculated as

(16) $$\mu_{t}=\frac{1}{N}\sum_{n=1}^{N}{\hat{y}}_{t}^{\left(n\right)},$$

and the predictive variance is calculated as

(17) $$\sigma_{t}^{2}=\frac{1}{N-1}\sum_{n=1}^{N}{\left({\hat{y}}_{t}^{\left(n\right)}-\mu_{t}\right)}^{2}.$$

A larger $\sigma_{t}^{2}$ indicates that the model is less certain about the current furnace condition. The corresponding prediction interval can be constructed as

(18) $$\left[\mu_{t}-\kappa \sigma_{t},\mu_{t}+\kappa \sigma_{t}\right],$$

where κ is determined by the desired confidence level. In this study, the 95% prediction interval is used for uncertainty evaluation.

### 2.6. Physics-Informed Monotonicity Constraint

Although the dual-task architecture improves representation learning under sparse supervision, a purely data-driven regression model may still produce responses that are inconsistent with the expected metallurgical behavior. According to the carbothermal reduction reaction in Equation (2), stronger reducing conditions generally promote the reduction of tin oxides and thereby decrease the residual tin content in the slag. Therefore, within the operating range represented by the training data, the predicted slag tin content is expected to exhibit a non-increasing relationship with the reducing condition.

Because the available process measurements do not include online ${\mathrm{CO}}_{2}$ concentration, slag composition, or thermodynamic activity data, a rigorous thermodynamic reduction metric cannot be calculated. Accordingly, an engineering-oriented raw reduction-condition proxy is defined for the input window ending at time *t* as

(19) $$R_{t}^{\mathrm{raw}}={\bar{\mathrm{CO}}}_{t}\;{\bar{\mathrm{Temp}}}_{t},$$

where ${\bar{\mathrm{CO}}}_{t}$ and ${\bar{\mathrm{Temp}}}_{t}$ denote the arithmetic means of the measured CO concentration and furnace temperature, respectively, over the input window $X_{t}$. The window means in Equation (19) are calculated in their original measurement scales before feature-wise normalization of the TCN inputs. CO concentration serves as an observable proxy for the reducing atmosphere, whereas furnace temperature represents the thermal conditions that affect reaction kinetics and mass transfer. Their product emphasizes the joint occurrence of a relatively high CO concentration and a relatively high operating temperature. Nevertheless, $R_{t}^{\mathrm{raw}}$ is only an engineering proxy and should not be interpreted as a rigorous thermodynamic quantity.

To remove the numerical scale of the raw product and prevent information leakage from the validation and testing periods, the proxy is standardized using statistics calculated exclusively from the chronological training period:

(20) $${\tilde{R}}_{t}=\frac{R_{t}^{\mathrm{raw}}-\mu_{R}^{\mathrm{tr}}}{s_{R}^{\mathrm{tr}}+\varepsilon_{R}},$$

where $\mu_{R}^{\mathrm{tr}}$ and $s_{R}^{\mathrm{tr}}$ are the mean and standard deviation, respectively, of $R^{\mathrm{raw}}$ over the training windows and $\varepsilon_{R}$ is a small positive constant introduced for numerical stability. Thus, ${\tilde{R}}_{t}$ is dimensionless and represents the deviation of the current reduction condition from the training-period mean in units of one training-period standard deviation. Because $s_{R}^{\mathrm{tr}}+\varepsilon_{R}>0$, this transformation preserves the sign of the local response relationship.

The shared temporal encoder extracts the latent process representation

(21) $$z_{t}=E_{\theta }\left(X_{t}\right),$$

where $E_{\theta }\left(\cdot \right)$ denotes the TCN encoder parameterized by θ. As defined in Equation (13), the standardized proxy ${\tilde{R}}_{t}$ is explicitly concatenated with $z_{t}$ before the resulting feature vector is passed to the regression head. This explicit computational connection makes the predicted slag tin content differentiable with respect to ${\tilde{R}}_{t}$.

The expected local monotonic relationship is formulated as

(22) $$g_{t}={\left.\frac{\partial {\hat{y}}_{t}}{\partial {\tilde{R}}_{t}}\right|}_{z_{t}}\leq 0,$$

where $g_{t}$ denotes the local sensitivity of the regression output to a one-standard-deviation increase in the reduction-condition proxy while the latent representation $z_{t}$ is held fixed. A positive value of $g_{t}$ indicates that the predicted slag tin content increases as the proxy becomes larger, which violates the prescribed non-increasing prior.

Accordingly, the physics-informed monotonicity loss is defined as

(23) $$L_{\mathrm{phy}}=\frac{1}{M}\sum_{i=1}^{M}\mathrm{ReLU}\left(g_{i}\right)=\frac{1}{M}\sum_{i=1}^{M}\max\left(\frac{\partial {\hat{y}}_{i}}{\partial {\tilde{R}}_{i}},0\right),$$

where *M* is the number of process windows in a mini-batch and $g_{i}$ is the local proxy-response gradient of the *i*-th window. The derivative is computed by automatic differentiation with ${\tilde{R}}_{i}$ retained in the computational graph. In implementation, the vector of per-window gradients is obtained from $\partial \sum_{i=1}^{M}{\hat{y}}_{i}/\partial \tilde{R}$ with higher-order gradient tracking enabled, so that $L_{\mathrm{phy}}$ contributes to parameter optimization.

Equation (23) penalizes only positive local gradients; therefore it encourages a non-increasing response without prescribing an exact functional form or response magnitude. Because the loss depends on the model output and the process-derived proxy rather than on laboratory slag assays, it can be applied to both labeled and unlabeled training windows. The constraint should be interpreted as a local directional regularizer within the observed operating domain rather than as a complete thermodynamic model.

### 2.7. Joint Optimization Strategy

The proposed PI-MTSS is trained through a joint optimization objective that combines supervised regression, self-supervised reconstruction, and physics-informed monotonicity regularization:

(24) $$L_{\mathrm{total}}=L_{\sup}+\lambda_{\mathrm{rec}}L_{\mathrm{rec}}+\lambda_{\mathrm{phy}}L_{\mathrm{phy}},$$

where $\lambda_{\mathrm{rec}}$ and $\lambda_{\mathrm{phy}}$ are weighting coefficients for the reconstruction loss and the physical loss, respectively.

When a mini-batch contains labeled samples, all three loss terms contribute to the parameter update. When slag labels are absent, the supervised loss is masked out by Equation (14), while the reconstruction loss and the physical loss continue to update the shared encoder and related modules. In this way, the proposed model can exploit the abundant high-frequency process data while still learning from the sparse slag assay labels.

The complete training procedure is summarized as follows:

1. Normalize the high-frequency process variables using the statistics of the training set.
2. Construct sliding window samples $X_{t}$ and align available slag tin labels using the label mask $m_{t}$.
3. Feed each input window into the shared TCN encoder to obtain the latent representation $z_{t}$.
4. Use the reconstruction branch to predict the future process state and calculate $L_{\mathrm{rec}}$.
5. Use the regression branch to predict slag tin content and calculate the masked supervised loss $L_{\sup}$.
6. Calculate the reduction-condition proxy index $R_{t}$ and the physics-informed monotonicity loss $L_{\mathrm{phy}}$.
7. Update all trainable parameters by minimizing $L_{\mathrm{total}}$.
8. During inference, perform multiple stochastic forward passes with MC Dropout to obtain the predictive mean and prediction interval.

## 3. Results

### 3.1. Dataset Characteristics and Intra-Month Variability

The dataset comprised a continuous 30-day production record collected from an industrial top-blown tin smelting furnace. This period was the only complete month for which continuous production records were retained and made available by the industrial partner. The period was selected before model development according to data availability, production continuity, and record completeness rather than predictive performance.

Four process measurements were used as model inputs: the temperature signal and the concentrations of CO, O_2_, and SO_2_. These measurements were synchronously recorded by the distributed control system at one-minute intervals, yielding 43,200 multivariate process time steps. Slag tin content was determined through offline laboratory assays approximately every three hours, providing 240 labeled observations. The high-frequency process records and sparse laboratory assays served different modeling purposes, with former characterizing the continuous evolution of the furnace state and the latter defining the supervised regression targets. No intermediate slag labels were generated through interpolation.

Table 1 provides a confidentiality-preserving overview of the dataset. Because the measurements originated from an operating industrial plant, the data use agreement with the industrial partner does not permit disclosure of the raw process variable values, actual engineering operating ranges, or complete time series. This restriction does not categorically prohibit the reporting of aggregate or grouped statistics; accordingly, the manuscript reports non-identifying summaries dthat are required in order to characterize the dataset and experimental design, including sample counts, acquisition frequencies, chronological split sizes, and aggregate model evaluation results. Only information that would reveal protected process values, actual engineering ranges, or reconstructable time-series trajectories is omitted. All model training, validation, and evaluation procedures were conducted using the original field measurements after preprocessing.

Missing and abnormal process records were handled using the fixed causality-preserving rules specified in Section 3.2. The slag assay sequence was not interpolated or artificially expanded. All data-dependent preprocessing parameters were estimated exclusively from the training period and were subsequently applied unchanged to the validation and testing periods.

To examine temporal variability within the available production month, the complete sequence was divided into six consecutive and non-overlapping five-day periods, denoted by P1–P6. The measured process signals and slag assay observations exhibited temporal fluctuations across these periods rather than remaining stationary throughout the month. The confidentiality restriction applies to the raw process variable values, actual engineering operating ranges, and complete time-series trajectories, and does not preclude the reporting of normalized, non-identifying, or performance-based grouped summaries. Thus, the chronological period definitions, sample counts, and model performance comparisons are reported, whereas information that could reveal protected process values or reconstruct the original time series is omitted.

The chronological organization was preserved in all subsequent experiments, and observations from later periods were not randomly mixed into the earlier training data. The rolling-origin evaluation presented in Section 3.4 was used to determine whether the model maintained its predictive performance across multiple unseen periods within the observed month. This performance-based evaluation provides a non-identifying assessment of intra-month temporal robustness without disclosing the protected raw process variable values, actual engineering operating ranges, or complete time-series trajectories. Nevertheless, because all observations originated from the same continuous production month, the results should not be interpreted as evidence of cross-month or long-term generalization.

### 3.2. Implementation Details and Experimental Protocol

All experiments were implemented in Python 3.10.13 using PyTorch 2.2.2, NumPy 1.26.4, and scikit-learn 1.4.2 with CUDA 12.1, and were executed on an NVIDIA GeForce RTX 4050 GPU (NVIDIA Corporation, Santa Clara, CA, USA). The 30-day sequence was divided chronologically into the first 20 days for training, the following 5 days for validation, and the final 5 days for testing. These periods contained 28,800, 7200, and 7200 one-minute process records and 160, 40, and 40 slag assays, respectively. The validation period was used for model selection and hyperparameter selection, with the testing period accessed only for final evaluation. Six-hour input windows containing 360 one-minute observations were constructed without using measurements later than the prediction time. For an assay near a partition boundary, its input window was allowed to include earlier measurements from the immediately preceding period because those measurements would have been available in an online setting; no future measurement or future assay was used. Only process windows ending within the training period were used to update the model parameters.

All four model inputs, namely temperature, CO, O_2_, and SO_2_, were original field measurements recorded by the distributed control system; none of the process variable sequences was simulated or generated for model training. After the records had been ordered by timestamp and aligned to the one-minute grid, duplicate timestamps were replaced by their variable-wise mean. Blank entries, non-finite values, and records carrying an invalid DCS quality flag were treated as missing. A gap of no more than five consecutive minutes was filled causally using the most recent valid observation. Any candidate input window containing a longer unresolved gap was excluded. Isolated impulsive spikes were detected independently for each process variable using a trailing 11-observation Hampel filter. A value was flagged when its absolute deviation from the trailing median exceeded three times the scaled median absolute deviation, where the scale factor was 1.4826; a flagged value was replaced by the corresponding trailing median. Sustained deviations were retained to avoid removing genuine changes in furnace operation. The slag assay values were neither interpolated nor used to replace missing process measurements. Finally, each process variable was standardized using the mean and standard deviation estimated from the training period, and the same parameters were applied unchanged to the validation and testing periods.

The shared TCN encoder comprised four residual temporal convolution blocks. Each block contained two causal one-dimensional convolutions followed by rectified linear unit activation and dropout. The output-channel widths of the four blocks were 64, 128, 128, and 128, and their dilation factors were 1, 2, 4, and 8, respectively. Both convolutions within a block used the block-specific dilation factor and a kernel size of 3. A 1×1 convolution was used in the residual path whenever the input and output channel dimensions differed. Adaptive temporal average pooling converted the last sequence feature map into a 128-dimensional latent vector $z_{t}$. The future state decoder was a multilayer perceptron with dimensions 128→64→4 and reconstructed the four normalized process variables at the next time step. For PI-MTSS, the standardized reduction-condition proxy index was concatenated with $z_{t}$, and the regression head used dimensions 129→64→32→1. Rectified linear unit activation was used between fully connected layers. A dropout probability of 0.20 was used after the temporal convolutions and hidden fully connected layers; the same dropout layers remained active during MC-dropout inference.

Model parameters were optimized using AdamW with $\beta_{1}=0.9$, $\beta_{2}=0.999$, a learning rate of $1\times 10^{-3}$, and a weight decay of $1\times 10^{-4}$. The total mini-batch size was 32. Each training mini-batch contained eight labeled assay-aligned windows and 24 unlabeled process windows sampled from the training period. The supervised loss was evaluated on the eight labeled windows, whereas the reconstruction and monotonicity losses were evaluated on all 32 windows. One epoch was defined as one pass through the 160 labeled training windows; the unlabeled windows were reshuffled and resampled without replacement within each epoch. Training was limited to 300 epochs. Early stopping was applied when the validation RMSE failed to improve for 30 consecutive epochs, and the checkpoint with the lowest validation RMSE was restored for evaluation. The loss coefficients were fixed at $\lambda_{\mathrm{rec}}=0.1$ and $\lambda_{\mathrm{phy}}=0.5$.

Table 2 summarizes the final configuration. The candidate learning rates were $\left\{10^{-4},5\times 10^{-4},10^{-3}\right\}$, the candidate weight decay values were $\left\{0,10^{-5},10^{-4}\right\}$, the candidate batch sizes were {16,32,64}, and the candidate dropout probabilities were {0.1,0.2,0.3}. Candidate settings were ranked by RMSE on the chronological validation period, with the testing period excluded from all selections. For each rolling-origin experiment, the same candidate sets and selection rule were used within that experiment’s own training and validation periods.

The SVR baseline received the same standardized six-hour windows as the neural models. Each 360×4 window was flattened into a 1440-dimensional vector. An RBF kernel was used, and the validation search considered C∈{1,10,100}, ϵ∈{0.01,0.05,0.1}, and $\gamma \in \{\mathrm{scale},10^{-3},10^{-2}\}$. The selected setting was C=10, ϵ=0.05, and γ=scale, where scale denotes the scikit-learn definition $1/(1440\;\mathrm{Var}\left(X_{\mathrm{train}}\right))$. The LSTM baseline contained two recurrent layers with 64 hidden units per layer and an inter-layer dropout probability of 0.20. Its final hidden state was passed through a 64→32→1 regression head. The validation search for LSTM considered one or two layers, hidden dimensions in {32,64,128}, and dropout probabilities in {0.1,0.2,0.3}. The Vanilla TCN used the same encoder and temporal aggregation operation as PI-MTSS, followed by a 128→64→32→1 regression head; it did not include the future state decoder, reduction-condition proxy input, or monotonicity loss. LSTM and Vanilla TCN were optimized using the same AdamW settings, mini-batch size, maximum epoch count, and early-stopping rule as PI-MTSS, but their batches contained only assay-aligned labeled windows.

The five prespecified neural-network seeds were 0, 1, 2, 3, and 4. Each seed was applied to Python PyTorch 2.2.2, NumPy 1.26.4, and PyTorch random-number generators before model initialization and data loader construction. The best validation checkpoint from each run was evaluated once on the 40 chronologically held-out test assays. Dropout was disabled for deterministic LSTM and Vanilla-TCN testing. For PI-MTSS, dropout remained active and 50 stochastic forward passes were performed; their mean was used for the point-prediction metrics, and their standard deviation was used to construct the MC-dropout epistemic uncertainty intervals. Neural model results were summarized as mean ± standard deviation across the five matched-seed runs. Because SVR is deterministic under fixed data and hyperparameters, it was fitted and evaluated once after validation-based hyperparameter selection.

For pairwise model comparisons, the predictions of each neural model were first averaged across the five matched random seeds at each of the 40 test assays, whereas the single deterministic SVR prediction sequence was retained unchanged. Temporal sampling uncertainty in the RMSE difference was estimated using a paired moving-block bootstrap with a block length of five consecutive assays and 10,000 bootstrap resamples. The 36 overlapping candidate blocks were constructed from the chronological testing sequence. For each bootstrap resample, eight blocks were sampled with replacement and concatenated to obtain 40 observations. The same resampled temporal indices were applied to the two methods being compared, and the 2.5th and 97.5th percentiles of the resulting RMSE-difference distribution formed the 95% confidence interval.

For the paired block-randomization test, the chronological testing sequence was partitioned into eight non-overlapping blocks of five consecutive assays. Within each block, the prediction sequences of the two compared methods were either retained or exchanged jointly while the measured slag values remained fixed. All $2^{8}=256$ possible blockwise assignments were enumerated. The two-sided exact *p*-value was calculated as the proportion of assignments satisfying

$$\left|\Delta_{\mathrm{RMSE}}^{*}\right|\geq \left|\Delta_{\mathrm{RMSE}}^{\mathrm{obs}}\right|.$$

The three pairwise *p*-values were subsequently adjusted using Holm’s procedure. Random seed 2026 was used for the moving-block bootstrap. Seed-to-seed variability and temporal resampling uncertainty were summarized separately, and the five training runs were not treated as five independent testing datasets.

For the uncertainty calibration analysis, the same paired moving-block bootstrap settings were applied to the 40 chronological test assays. Within each bootstrap resample, identical temporal indices were used across all nominal coverage levels and all five random seeds. Empirical coverage was calculated separately for each seed and then averaged across seeds. At each nominal coverage level, the 2.5th and 97.5th percentiles of the 10,000 bootstrap estimates formed the pointwise 95% confidence band. Where a confidence interval for Spearman’s $\rho_{s}$ was reported, the coefficient was recalculated within every bootstrap resample using the same resampled temporal indices.

### 3.3. Main Comparison and Statistical Significance

The proposed PI-MTSS was compared with support vector regression (SVR), LSTM, and Vanilla TCN using the same chronologically held-out testing period. The testing set contained the final 40 laboratory assays from the observed production month. All preprocessing parameters and model hyperparameters were determined using only the training and validation periods, whereas the testing period was used exclusively for final evaluation.

To quantify the variability introduced by model initialization and stochastic optimization, each neural model was independently trained using five common random seeds. For PI-MTSS, the predictive mean obtained from 50 MC dropout forward passes was used to calculate the point prediction metrics. Because SVR is deterministic under fixed data and hyperparameters, it was trained and evaluated once using the same chronological partition and preprocessing procedure. Table 3 reports the root mean square error (RMSE), mean absolute error (MAE), and coefficient of determination ($R^{2}$). The results of the neural models are expressed as mean ± standard deviation across the five runs.

PI-MTSS achieved the lowest prediction errors and highest $R^{2}$ among the evaluated models, obtaining a mean RMSE of 0.198, mean MAE of 0.145, and mean $R^{2}$ of 0.889. Among the comparison methods, Vanilla TCN provided the strongest baseline performance, with an RMSE of 0.215±0.005, MAE of 0.158±0.004, and $R^{2}$ of 0.869±0.006. Relative to Vanilla TCN, PI-MTSS reduced RMSE and MAE by 7.91% and 8.23%, respectively, while increasing $R^{2}$ by 0.020. PI-MTSS obtained a lower RMSE than Vanilla TCN in all five matched-seed runs.

Compared with LSTM, PI-MTSS reduced RMSE from 0.248 to 0.198 and MAE from 0.186 to 0.145, corresponding to relative reductions of 20.16% and 22.04%, respectively. The corresponding increase in $R^{2}$ was 0.063. Compared with SVR, PI-MTSS reduced RMSE and MAE by 36.54% and 40.82%, respectively, and increased $R^{2}$ by 0.165.

The standard deviations of RMSE, MAE, and $R^{2}$ obtained by PI-MTSS were 0.004, 0.003, and 0.005, respectively. The corresponding standard deviations for Vanilla TCN were 0.005, 0.004, and 0.006. Thus, PI-MTSS exhibited slightly lower run-to-run variability than the strongest baseline for all three evaluation metrics.

Pairwise statistical comparisons were conducted using the seed-averaged prediction sequence of each neural model on the same 40 chronological test assays. The five random seeds were used to characterize initialization and optimization variability, and were not treated as independent testing datasets. In accordance with the procedure specified in Section 3.2, temporal dependence among consecutive assays was accounted for using a paired moving-block bootstrap and an exact paired block-randomization test. The primary comparison statistic was the RMSE difference:

(25) $$\Delta_{\mathrm{RMSE}}={\mathrm{RMSE}}_{\mathrm{baseline}}-{\mathrm{RMSE}}_{\mathrm{PI-MTSS}},$$

where a positive value indicates a lower RMSE for PI-MTSS. The corresponding relative RMSE reduction was calculated as

(26) $$\Delta_{\mathrm{RMSE}}^{\left(\%\right)}=\frac{{\mathrm{RMSE}}_{\mathrm{baseline}}-{\mathrm{RMSE}}_{\mathrm{PI-MTSS}}}{{\mathrm{RMSE}}_{\mathrm{baseline}}}\times 100\%.$$

Table 4 reports the pairwise RMSE differences, relative reductions, 95% temporal block-bootstrap confidence intervals, and adjusted *p*-values. The *p*-values obtained from the paired block-permutation tests were adjusted using the Holm procedure to account for the three baseline comparisons.

Relative to SVR, PI-MTSS produced an RMSE difference of 0.114, with a 95% confidence interval of [0.076,0.151] and a Holm-adjusted *p*-value of 0.006. Relative to LSTM, the RMSE difference was 0.050, with a 95% confidence interval of [0.018,0.080] and a Holm-adjusted *p*-value of 0.024. Both confidence intervals excluded zero, indicating statistically significant improvements at the 0.05 level.

For the comparison with Vanilla TCN, the RMSE difference was 0.017, corresponding to a relative reduction of 7.91%. Its 95% confidence interval was [−0.002,0.036] and the Holm-adjusted *p*-value was 0.094. Although PI-MTSS obtained a lower mean RMSE in all five matched-seed runs, the confidence interval included zero and the difference did not reach statistical significance after correction for multiple comparisons. Overall, two of the three pairwise comparisons remained statistically significant after Holm correction.

Only aggregate model performance and statistical quantities are reported in this subsection. Sample-level prediction trajectories, measured slag values, and predicted-versus-measured scatter plots are omitted in order to comply with industrial confidentiality requirements.

### 3.4. Rolling-Window Temporal Validation

Although the chronologically held-out test set used in Section 3.3 evaluates the models on the final production period, the result from a single split may be affected by the temporal characteristics of that particular period. Therefore, a rolling-origin temporal validation procedure was further conducted in order to examine whether the relative performance of the models remained stable across multiple unseen periods within the observed production month.

The six consecutive five-day periods defined in Section 3.1 were used to construct three expanding-window evaluations, as summarized in Table 5. Each period contained 40 laboratory assay observations. In the first window, P1–P2 were used for training, P3 was used for validation, and P4 was used for testing. The training period was then progressively expanded in the subsequent windows, while the validation and testing periods were shifted forward chronologically. The third window was identical to the final chronological partition used in Section 3.3.

For every window, each model was retrained from scratch. Normalization parameters and other data-dependent preprocessing quantities were estimated exclusively from the corresponding training period. Hyperparameter selection and early stopping were performed using only the immediately subsequent validation period. Neither the labeled observations nor the high-frequency process sequences from the testing period were used for model training, self-supervised reconstruction, or model selection. Consequently, all predictions were generated for chronologically later and previously unseen observations.

The neural models were evaluated using the same five random seeds as in the main comparison. For PI-MTSS, the predictive mean obtained from 50 MC dropout forward passes was used to calculate the point prediction metrics. Table 6 reports the seed-averaged RMSE, MAE, and $R^{2}$ for each temporal window. The summary rows show the mean and standard deviation across the three testing periods. These across-window standard deviations describe temporal performance variation, and should not be interpreted as confidence intervals.

PI-MTSS achieved the lowest RMSE and MAE and the highest $R^{2}$ in all three testing periods. Its RMSE ranged from 0.190 to 0.210, while its MAE ranged from 0.139 to 0.155 and its $R^{2}$ from 0.872 to 0.900; the corresponding across-window averages were 0.199, 0.146, and 0.887, respectively.

Vanilla TCN remained the strongest baseline in each temporal window, obtaining an average RMSE of 0.217, average MAE of 0.160, and average $R^{2}$ of 0.865. Compared with Vanilla TCN, the fold-wise RMSE reductions achieved by PI-MTSS were 8.30%, 8.21%, and 7.91% in W1, W2, and W3, respectively, for a mean fold-wise RMSE reduction of 8.14%; the mean fold-wise MAE reduction was 8.53%, while the average improvement in $R^{2}$ was 0.022.

Temporal variation was observed for all models, with W1 producing the highest prediction error, W2 the lowest error, and W3 moderately increased error. Nevertheless, the ranking of the four methods remained unchanged across the three windows. Moreover, the across-window standard deviations obtained by PI-MTSS were slightly lower than those of Vanilla TCN for all three metrics, indicating that its numerical performance advantage was maintained across the evaluated intra-month periods.

The rolling windows contained overlapping historical training data, and should not be treated as statistically independent experimental replicates; accordingly, the across-window results are used as descriptive evidence of temporal robustness rather than for additional significance testing. Furthermore, because all validation windows originated from the same continuous production month, these results only demonstrate intra-month temporal generalization, not cross-month, cross-furnace, or long-term deployment performance.

### 3.5. Component Ablation and Interaction Analysis

A full component ablation experiment was conducted to distinguish the individual and joint contributions of the three main components of PI-MTSS. The investigated factors were defined as follows: factor A denotes the self-supervised future-state reconstruction task, factor B denotes the physics-informed monotonicity constraint, and factor C denotes MC dropout inference. The resulting $2^{3}$ factorial design included the baseline and all seven possible component combinations.

All configurations were evaluated using the same chronological partition as adopted in Section 3.3, corresponding to W3 in Section 3.4. For each combination of A and B, the model was independently trained using the same five random seeds. The presence or absence of C represents an inference-mode difference rather than a separate training objective. Specifically, all models used the same dropout structure during training. When C was disabled, dropout was deactivated during testing and a single deterministic forward pass was performed. When C was enabled, dropout remained active and the predictive mean of 50 stochastic forward passes was used as the point prediction.

In addition to RMSE, MAE, and $R^{2}$, the configurations using MC dropout were evaluated using the prediction interval coverage probability at 90% nominal coverage (PICP_90_), the corresponding normalized mean prediction interval width (NMPIW_90_), and the average calibration error (ACE). NMPIW_90_ was calculated as

(27) $${\mathrm{NMPIW}}_{90}=\frac{1}{N_{\mathrm{test}}R_{y}}\sum_{i=1}^{N_{\mathrm{test}}}\left(U_{i}^{\left(0.90\right)}-L_{i}^{\left(0.90\right)}\right),$$

where $L_{i}^{\left(0.90\right)}$ and $U_{i}^{\left(0.90\right)}$ denote the lower and upper bounds of the 90% prediction interval, respectively, and $R_{y}$ is the range of the slag tin content calculated using the training period only. The calibration error was evaluated over the nominal coverage set Γ={0.50,0.60,0.70,0.80,0.90,0.95}:

(28) $$\mathrm{ACE}=\frac{1}{\left|\Gamma \right|}\sum_{\gamma \in \Gamma }\left|{\mathrm{PICP}}_{\gamma }-\gamma \right|.$$

A lower ACE indicates closer agreement between empirical and nominal interval coverage. The uncertainty metrics are not defined for the deterministic configurations without C.

The complete PI-MTSS obtained an RMSE of 0.198±0.004, MAE of 0.145±0.003, and $R^{2}$ of 0.889±0.005. Relative to V0, these values corresponded to a reduction of 7.91% in RMSE, a reduction of 8.23% in MAE, and an increase of 0.020 in $R^{2}$. Under deterministic inference, V1 and V2 obtained RMSE values of 0.206 and 0.211, respectively, compared with 0.215 for V0; for V3, which used the MC dropout predictive mean without A or B, an RMSE of 0.213 was obtained.

Among the reduced configurations, V4 obtained an RMSE of 0.200, MAE of 0.147, and $R^{2}$ of 0.886; the corresponding values for V7 were 0.198, 0.145, and 0.889. The RMSE difference between V7 and V4 was 0.002, with a paired moving-block-bootstrap 95% confidence interval of [−0.001,0.005].

To quantify whether A and B produced additive, complementary, or redundant effects, a difference-in-differences interaction measure was calculated. For a lower-is-better error metric *E*, the interaction under inference mode C=c was defined as

(29) $$I_{\mathrm{AB},c}^{\left(E\right)}=\left(E_{00c}-E_{11c}\right)-\left[\left(E_{00c}-E_{10c}\right)+\left(E_{00c}-E_{01c}\right)\right],$$

where $E_{abc}$ denotes the metric obtained when A, B, and C take the binary states *a*, *b*, and *c*, respectively. A positive value indicates that the joint error reduction exceeds the sum of the two individual reductions. For $R^{2}$, the signs were arranged such that a positive value likewise indicates an improvement beyond additivity:

(30) $$I_{\mathrm{AB},c}^{\left(R^{2}\right)}=\left(R_{11c}^{2}-R_{00c}^{2}\right)-\left[\left(R_{10c}^{2}-R_{00c}^{2}\right)+\left(R_{01c}^{2}-R_{00c}^{2}\right)\right].$$

The three-factor interaction contrast was subsequently calculated as

(31) $$I_{\mathrm{ABC}}=I_{\mathrm{AB},1}-I_{\mathrm{AB},0}.$$

According to Table 7, the A–B interaction quantities were 0.002 for the reduction in RMSE under both inference modes, $I_{\mathrm{AB},0}=0.001$ and $I_{\mathrm{AB},1}=0.000$ for the reduction in MAE, and 0.005 and 0.004 for the increase in $R^{2}$. The three-factor contrasts ranged from −0.001 to 0.000.

For the uncertainty metrics, as shown in Table 8, V3 obtained a PICP_90_ of 82.0%, NMPIW_90_ of 0.252, and ACE of 0.071. The corresponding values were 87.5%, 0.229, and 0.039 for V5; 85.0%, 0.238, and 0.052 for V6; and 90.5%, 0.211, and 0.021 for V7. Relative to V3, V7 increased PICP_90_ by 8.5 percentage points and reduced NMPIW_90_ and ACE by 16.27% and 70.42%, respectively. The functional interpretation of these component-wise changes is provided in Section 4.2.

### 3.6. Analysis of Physical Consistency and Reduction Potential

The physical consistency of each model was evaluated on the same 40 chronologically held-out assay observations used in the preceding experiments. For each testing window, the reduction potential index was calculated according to the definition introduced in the Section 2.6:

(32) $$R_{i}={\bar{\mathrm{CO}}}_{i}\;{\bar{\mathrm{Temp}}}_{i},$$

where ${\bar{\mathrm{CO}}}_{i}$ and ${\bar{\mathrm{Temp}}}_{i}$ denote the mean CO concentration and mean furnace temperature, respectively, within the corresponding six-hour input window. To remove the influence of the numerical scale of $R_{i}$, the index was standardized using only the training-period statistics:

(33) $${\tilde{R}}_{i}=\frac{R_{i}-\mu_{R}^{\mathrm{tr}}}{s_{R}^{\mathrm{tr}}+\varepsilon },$$

where $\mu_{R}^{\mathrm{tr}}$ and $s_{R}^{\mathrm{tr}}$ are the mean and standard deviation of the reduction potential index in the training period. Because $s_{R}^{\mathrm{tr}}>0$, this transformation does not change the sign of the physical response.

For the deterministic configurations, the response gradient was calculated from a single test-time forward pass. For the configurations using MC dropout, the predictive mean of 50 stochastic forward passes was used. The local response gradient of the *i*-th testing sample under the *s*-th random seed was defined as

(34) $$g_{i}^{\left(s\right)}=\frac{\partial {\bar{y}}_{i}^{\left(s\right)}}{\partial {\tilde{R}}_{i}},$$

where ${\bar{y}}_{i}^{\left(s\right)}$ denotes either the deterministic prediction or the MC-dropout predictive mean. According to the expected inverse relationship between reduction-condition proxy index and residual slag tin content, $g_{i}^{\left(s\right)}\leq 0$ represents a physically consistent response, whereas $g_{i}^{\left(s\right)}>0$ represents a physical violation.

Two quantitative indicators were used to evaluate the gradient responses. The physical violation rate (PVR) and mean positive gradient magnitude (MPG) were calculated as

(35) $${\mathrm{PVR}}^{\left(s\right)}=\frac{100}{N_{\mathrm{test}}}\sum_{i=1}^{N_{\mathrm{test}}}1\left(g_{i}^{\left(s\right)}>\epsilon_{g}\right),$$

(36) $${\mathrm{MPG}}^{\left(s\right)}=\frac{1}{N_{\mathrm{test}}}\sum_{i=1}^{N_{\mathrm{test}}}\max\left(g_{i}^{\left(s\right)},0\right),$$

where $N_{\mathrm{test}}=40$, 1(·) is the indicator function, and $\epsilon_{g}=10^{-6}$ is a numerical tolerance. A lower PVR indicates fewer physically inconsistent samples, while a lower MPG indicates a smaller overall magnitude of the remaining violations.

**Figure 3:**
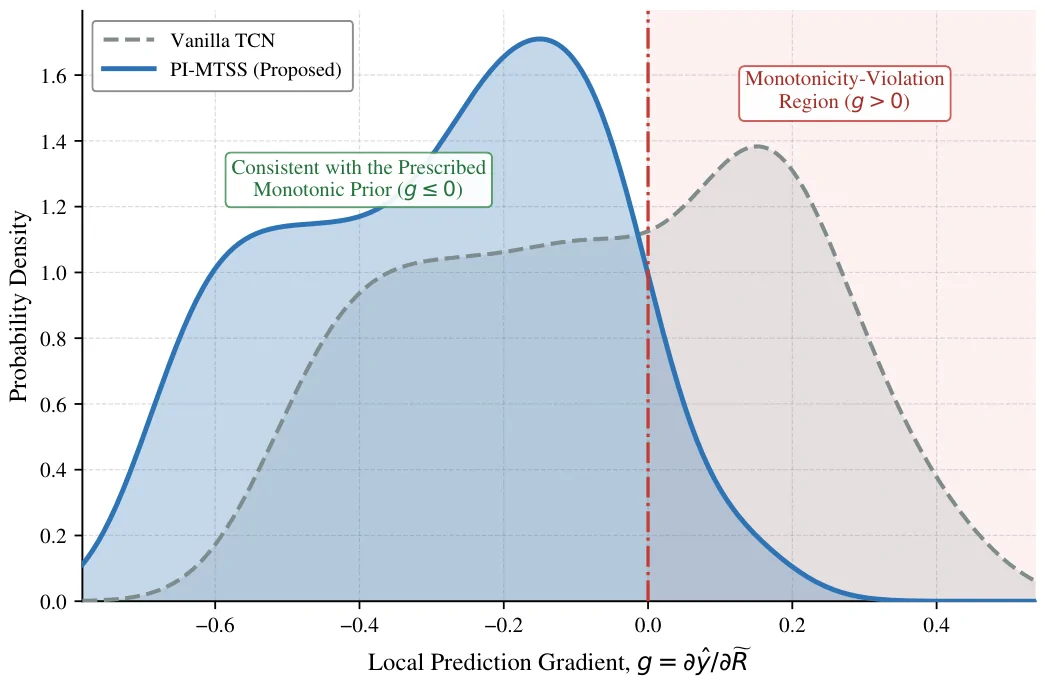
Distributions of the prediction gradients with respect to the standardized reduction potential index for the base TCN and PI-MTSS. The kernel density curves pool the gradients of the 40 testing windows obtained under five random seeds. Positive gradients indicate physically inconsistent responses, whereas non-positive gradients satisfy the expected inverse relationship between reduction-condition proxy index and residual slag tin content.

Figure 3 shows the gradient distributions of the Base TCN and complete PI-MTSS. The Base TCN distribution extended across both sides of zero and contained a substantial positive gradient region. In comparison, the PI-MTSS gradients were concentrated in the non-positive region, with only a small residual positive tail.

Table 9 reports the quantitative physical consistency results for all eight component configurations defined in Section 3.5. The gradients are expressed in units of slag tin content in wt.% per one training-period standard deviation of the reduction potential index.

Among the configurations without the physical constraint, the PVR ranged from 32.0% to 42.5% and the MPG ranged from 0.055 to 0.083. After factor B was enabled, the PVR decreased to between 3.5% and 8.0%, while the MPG decreased to between 0.004 and 0.010.

For the four matched comparisons V0–V2, V1–V4, V3–V6, and V5–V7, introducing the physical constraint reduced the PVR by 34.5, 30.0, 33.5, and 28.5 percentage points, respectively, for an average absolute reduction of 31.6 percentage points. The corresponding relative reductions in MPG were 88.0%, 90.2%, 89.7%, and 92.7%, with an average reduction of 90.1%.

The complete PI-MTSS obtained a PVR of 3.5±1.4%, compared with 42.5±4.7% for the base TCN. Its MPG decreased from 0.083±0.011 to 0.004±0.001, corresponding to a relative reduction of 95.2%. In addition, the median gradient shifted from −0.041 to −0.257, and the 95th-percentile gradient shifted from 0.331 to −0.009. Thus, at least 95% of the gradient responses of the complete model were non-positive after aggregation over the evaluated random seeds.

To examine whether the local gradient behavior was also reflected in the observed operating samples, the 40 testing windows were ordered according to ${\tilde{R}}_{i}$ and divided into low, medium, and high reduction potential groups using the empirical 33rd and 67th percentiles of the testing set. This grouping was performed only for post hoc evaluation, and was not used for model training, hyperparameter selection, or threshold determination.

According to Table 10, the measured mean slag tin content decreased from 2.35 wt.% in the low reduction potential group to 1.31 wt.% in the high reduction potential group, corresponding to a decrease of 1.04 wt.%. The Base TCN predicted a smaller decrease from 2.16 to 1.50 wt.%, whereas PI-MTSS predicted a decrease from 2.30 to 1.35 wt.%.

A linear trend weighted by sample count was fitted to the three group means:

(37) $$\beta_{m}=\frac{\sum_{q=1}^{3}n_{q}\left({\bar{R}}_{q}-\bar{R}\right)\left({\bar{y}}_{m,q}-{\bar{y}}_{m}\right)}{\sum_{q=1}^{3}n_{q}{\left({\bar{R}}_{q}-\bar{R}\right)}^{2}},$$

where *m* denotes either the measured value or model prediction and *q* denotes the reduction potential group. The measured data produced a slope of −0.561 wt.% per standard deviation of the reduction-condition proxy index. The corresponding slopes were −0.357 for the base TCN and −0.513 for PI-MTSS; therefore, the absolute deviation from the measured slope decreased from 0.204 for the base TCN to 0.048 for PI-MTSS.

The sample-weighted aggregation of the three group RMSE values was 0.215 for the base TCN and 0.198 for PI-MTSS, consistent with the overall testing results reported in the preceding sections. Across all three reduction potential levels, PI-MTSS obtained a lower group-wise RMSE than the base TCN. Therefore, the local gradient analysis and the reduction potential grouping analysis produced consistent numerical results, with PI-MTSS exhibiting fewer positive-gradient responses and a decreasing group-level trend closer to that observed in the laboratory measurements.

### 3.7. Uncertainty Calibration and Reliability

The calibration and reliability of the uncertainty estimates were evaluated using the same 40 chronologically held-out assay observations employed in the preceding experiments. For each of the five random seeds, the model was evaluated once on the fixed testing set, and 50 stochastic forward passes were performed with dropout activated. No testing sample was used for model selection, interval scaling, calibration parameter estimation, or threshold determination.

Only the four configurations containing factor C were included in this analysis, since the deterministic configurations did not directly generate uncertainty intervals. As defined in Section 3.5, these configurations were V3 (C), V5 (A+C), V6 (B+C), and V7 (A+B+C). Factors A, B, and C denote self-supervised future state reconstruction, physics-informed monotonicity regularization, and MC dropout inference, respectively.

For a nominal coverage level γ, the uncertainty interval of the *i*-th testing sample was constructed as

(38) $$I_{i}^{\left(\gamma \right)}=\left[\mu_{i}-\kappa_{\gamma }\sigma_{i},\;\mu_{i}+\kappa_{\gamma }\sigma_{i}\right],$$

where $\mu_{i}$ and $\sigma_{i}$ are the mean and standard deviation of the 50 stochastic predictions, respectively, and

(39) $$\kappa_{\gamma }=\Phi^{-1}\left(\frac{1+\gamma }{2}\right)$$

is the corresponding standard-normal quantile. The nominal coverage levels were

(40) $$\Gamma =\left\{0.50,0.60,0.70,0.80,0.90,0.95\right\}.$$

Because the interval in Equation (38) was obtained from stochastic model variation without adding a separate observation noise term, it is interpreted as an MC-dropout epistemic uncertainty interval rather than a complete probabilistic description of all industrial measurement uncertainties.

**Figure 4:**
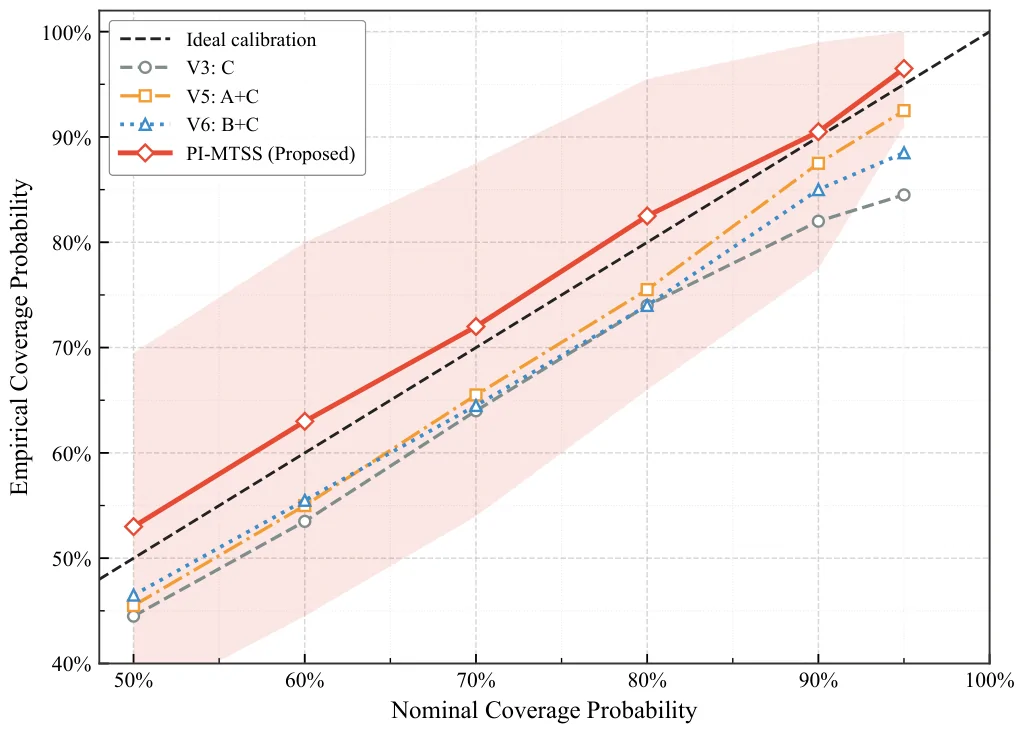
Nominal versus empirical interval coverage on the chronologically held-out testing set. The diagonal line denotes ideal calibration, and each calibration curve represents the mean result over five random seeds. The shaded region for PI-MTSS denotes the pointwise 95% confidence band obtained from 10,000 paired moving-block bootstrap resamples with a block length of five consecutive assays, as specified in Section 3.2.

Figure 4 compares the nominal and empirical coverage probabilities. The diagonal line represents ideal calibration. For the complete PI-MTSS, the empirical coverage probabilities at nominal levels of 50%, 60%, 70%, 80%, 90%, and 95% were 53.0%, 63.0%, 72.0%, 82.5%, 90.5%, and 96.5%, respectively.

The calibration curve of V3 remained below the ideal diagonal at all evaluated levels. Its empirical coverage probabilities at the 90% and 95% nominal levels were 82.0% and 84.5%, respectively. Adding factor A increased these two coverage probabilities to 87.5% and 92.5%, whereas adding factor B increased them to 85.0% and 88.5%. When factors A and B were both combined with MC dropout, the empirical curve was closest to the ideal diagonal over the complete range of nominal coverage levels.

Table 11 summarizes the interval calibration, interval sharpness, and error–uncertainty consistency results. The normalized mean prediction interval widths were calculated using the slag tin content range obtained exclusively from the training period. All values are reported as mean ± standard deviation over five random seeds.

At the 90% nominal level, PI-MTSS obtained an empirical coverage of 90.5±1.4%, NMPIW of 0.211±0.006, and absolute coverage deviation of 0.5 percentage points. At the 95% nominal level, the corresponding values were 96.5±1.4%, 0.245±0.007, and 1.5 percentage points. Its ACE across the six nominal levels was 0.021±0.004. Relative to V3, PI-MTSS reduced NMPIW_90_ and NMPIW_95_ by 16.27% and 24.62%, respectively, and reduced ACE by 70.42%.

To examine whether the estimated uncertainty identified samples with larger prediction errors, the absolute prediction error was defined as

(41) $$a_{i}=\left|y_{i}-\mu_{i}\right|,$$

and its rank association with the MC-dropout standard deviation was measured using

(42) $$\rho_{s}=\mathrm{corr}\left[\mathrm{rank}\left(\sigma_{i}\right),\mathrm{rank}\left(a_{i}\right)\right].$$

All four MC dropout configurations produced positive uncertainty–error correlations. V3 obtained a Spearman coefficient of 0.438±0.055, whereas the coefficients of V5 and V6 were 0.612±0.047 and 0.548±0.051, respectively. The complete PI-MTSS obtained the highest coefficient of 0.691±0.042.

For a complementary group-level analysis, the 40 testing observations were sorted according to $\sigma_{i}$ and divided into four uncertainty quartiles, each containing ten observations. For PI-MTSS, the mean absolute errors from the quartile with the lowest uncertainty to the one with the highest were as follows:

(43) $$\left[0.078\pm 0.006,\;0.113\pm 0.007,\;0.157\pm 0.009,\;0.232\pm 0.012\right].$$

The mean absolute error increased across the four ordered uncertainty groups. The quartile with the highest uncertainty had an MAE 2.97 times that of the lowest-uncertainty quartile, and the equally weighted mean of the four quartile-level MAEs was 0.145. Among the four MC dropout configurations, V7 had the lowest ACE and normalized interval widths along with the highest Spearman coefficient. The interpretation and operational scope of these metrics are discussed in Section 4.4.

### 3.8. Hyperparameter Sensitivity

The sensitivity of PI-MTSS to the main model and inference hyperparameters was evaluated using the chronologically preceding validation period (P5). The earlier periods (P1–P4) were used for model training, whereas the final testing period (P6) was not involved in hyperparameter selection. Each candidate configuration was trained independently using five random seeds, and the results are reported as mean ± standard deviation.

The analysis considered the input window length *T*, reconstruction loss coefficient $\lambda_{\mathrm{rec}}$, monotonicity loss coefficient $\lambda_{\mathrm{phy}}$, and relative contributions of CO concentration and furnace temperature to the reduction potential index. A one-factor-at-a-time protocol was adopted: when one hyperparameter was varied, the remaining hyperparameters were fixed at their default values,

(44) $$T=6\;h,\;\lambda_{\mathrm{rec}}=0.1,\;\lambda_{\mathrm{phy}}=0.5.$$

The learning rate was fixed at $1\times 10^{-3}$ for all configurations. Prediction accuracy was evaluated using RMSE, MAE, and $R^{2}$. Physical consistency was evaluated using the physical violation rate (PVR) and mean positive gradient magnitude (MPG) defined in Section 3.6.

To examine the sensitivity of the reduction potential formulation without changing its physical direction, a dimensionless exponent perturbation form was introduced only for this diagnostic experiment:

(45) $$R_{i}^{\left(a,b\right)}={\left(\frac{{\bar{\mathrm{CO}}}_{i}}{\mu_{\mathrm{CO}}^{\mathrm{tr}}}\right)}^{a}{\left(\frac{{\bar{\mathrm{Temp}}}_{i}}{\mu_{\mathrm{Temp}}^{\mathrm{tr}}}\right)}^{b},\;a+b=2,$$

where $\mu_{\mathrm{CO}}^{\mathrm{tr}}$ and $\mu_{\mathrm{Temp}}^{\mathrm{tr}}$ are the corresponding means calculated from the training period. Each candidate index was subsequently standardized using its own training period statistics:

(46) $${\tilde{R}}_{i}^{\left(a,b\right)}=\frac{R_{i}^{\left(a,b\right)}-\mu_{R}^{\mathrm{tr},(a,b)}}{s_{R}^{\mathrm{tr},(a,b)}+\varepsilon }.$$

The default setting (a,b)=(1.0,1.0) is proportional to the original product of the mean CO concentration and mean furnace temperature. Consequently, after Z-score standardization, it is equivalent to the reduction potential index used in the complete PI-MTSS. Based on the content presented in Table 12, we can draw the following conclusions.

For the input window experiment, the lowest validation RMSE and MAE were obtained at T=6 h, with values of 0.201±0.005 and 0.147±0.004, respectively. The RMSE values at 2, 4, 8, and 10 h were 0.216, 0.206, 0.204, and 0.210. The corresponding PVR values ranged from 4.0% to 8.5%.

For $\lambda_{\mathrm{rec}}$, the validation RMSE decreased from 0.209 at 0 to 0.201 at 0.1 and increased to 0.211 at 0.5. The corresponding MAE values were 0.153, 0.147, and 0.155. For $\lambda_{\mathrm{phy}}$, increasing the coefficient from 0 to 2.0 reduced PVR from 31.5±4.1% to 1.0±0.9% and MPG from 0.056±0.008 to 0.001±0.001. The lowest validation RMSE in this series was 0.201±0.005 at $\lambda_{\mathrm{phy}}=0.5$; the RMSE at $\lambda_{\mathrm{phy}}=2.0$ was 0.212±0.008.

Across the five exponent pairs, the validation RMSE ranged from 0.201 to 0.205 and the PVR ranged from 4.0% to 6.0%. The default pair (a,b)=(1.0,1.0) obtained the lowest mean RMSE and MPG in this experiment. The mechanisms associated with these sensitivity patterns are discussed in Section 4.3 and Section 4.5.

The number of MC dropout forward passes *N* does not affect model training, but does determine the stability and computational cost of uncertainty estimation. Its sensitivity was evaluated separately while keeping the trained model parameters unchanged, adopting the same nominal coverage levels used in Section 3.7.

Increasing *N* from 10 to 50 changed PICP_95_ from 89.5% to 95.5%, ACE from 0.051 to 0.022, and the uncertainty–error Spearman coefficient from 0.604 to 0.687. Increasing *N* from 50 to 100 left PICP_95_ unchanged, changed NMPIW_95_ from 0.246 to 0.247, reduced ACE from 0.022 to 0.021, and increased $\rho_{s}$ from 0.687 to 0.692. The relative sampling cost increased from 1.00 to 2.00. The default settings in Equation (44), exponent pair (1.0,1.0), and N=50 were fixed for the chronologically held-out testing period (Table 13).

### 3.9. Computational Cost and Online Applicability

The computational cost of PI-MTSS was evaluated on the same local workstation used for model development, which was equipped with an NVIDIA GeForce RTX 4050 GPU. The model was implemented in PyTorch. Computational complexity was calculated using a single input window of 360 one-minute process samples. For latency evaluation, the batch size was fixed to one to simulate sample-by-sample online deployment. Before timing, 100 warm-up runs were performed. The reported latency was then calculated from 1000 inference runs in each of five independent repetitions, with CUDA synchronization applied before and after each timed run. The measured end-to-end model latency included input normalization, rolling window update, host-to-device transfer, stochastic forward propagation, and uncertainty aggregation.

The reconstruction decoder and the monotonicity loss calculation are required only during offline training. During online deployment, the reconstruction branch can be removed, with only the shared temporal encoder, regression head, and MC dropout sampling procedure retained. Table 14 summarizes the resulting model complexity. The complete training graph contained approximately 0.318 million trainable parameters, whereas the deployed prediction graph involved approximately 0.304 million active parameters. Thus, the training-only reconstruction decoder accounted for approximately 4.4% of the total parameters. The monotonicity constraint and MC dropout introduced no additional trainable parameters.

Table 15 reports the measured latency for different sampling numbers. A deterministic single-pass prediction required 1.3±0.1 ms per window. At N=50, the complete uncertainty-aware prediction required 58.4±1.2 ms, with a 95th-percentile latency of 60.5 ms. At N=100, the corresponding values were 116.2±2.1 ms and 119.8 ms.

At the selected setting of N=50, the mean inference latency occupied 0.097% of the 60-s process data update interval. Increasing the sampling number from 50 to 100 changed the update budget occupation from 0.097% to 0.194%.

The 6-h input window does not imply a 6-h prediction delay; it is maintained as a rolling buffer containing the most recent 360 process observations. After the buffer has been initialized, the oldest observation is discarded and the newest measurement is appended whenever the process variables are updated, allowing a new slag tin-content estimate to be generated every minute. Moreover, neither a current laboratory assay nor execution of the reconstruction branch is required during inference, with newly obtained assay results needed only for subsequent performance evaluation, recalibration, or periodic model updating.

The timing experiment measured the soft sensor model on the evaluated workstation. Industrial DCS communication, database access, visualization, network delays, and closed-loop actuator execution were not included. The engineering interpretation of the measured model-side latency is provided in Section 4.5.

## 4. Discussion

### 4.1. Predictive Performance and Temporal Robustness

The main comparison and rolling origin results in Table 3 and Table 6 show a consistent numerical ranking across the evaluated chronological periods. This pattern can be interpreted from the different ways in which the models use temporal and supervisory information. SVR receives each aligned sequence as a fixed-dimensional vector, and does not explicitly learn a hierarchy of delayed temporal features. LSTM and Vanilla TCN model sequence structure, but their regression objectives depend directly on the sparse slag assay labels.

The supervisory imbalance is substantial: dense process measurements describe the continuous furnace trajectory, whereas only the assay observations provide direct slag tin targets. PI-MTSS does not create additional target labels; instead, it uses the dense process stream to learn temporal structure through an auxiliary task and uses a directional process prior to regularize the sparse-label regression mapping. The observed performance tendency is consistent with more effective use of information already present in the training period rather than with an increase in the number of independent supervised samples. Overlapping windows and adjacent process records remain temporally correlated, and must not be counted as independent quality labels.

The comparison with Vanilla TCN nevertheless requires conservative wording. Although the direction of the effect was consistent across the reported point prediction metrics and matched-seed runs, the paired comparison did not reach the conventional significance level. This does not establish equivalence; rather, it indicates that the current test sequence is too limited to support a conclusive superiority claim over the strongest baseline. Accordingly, the effect size, confidence interval, and adjusted paired-test result should be considered jointly, and the phrase “significantly outperformed” is not warranted for this comparison.

The rolling-origin experiment extends the assessment beyond one terminal split and shows that the model ranking was retained as the evaluation period moved forward. The remaining variation among windows is compatible with a time-varying industrial process and motivates continued performance monitoring and periodic updating. Because the rolling windows share historical data and all originate from one furnace during one continuous month, they provide within-month temporal evidence only, and do not establish robustness across months, furnaces, feedstock batches, maintenance cycles, or long-term production regimes.

### 4.2. Complementary Roles and Interactions of Model Components

The ablation patterns in Table 7 and Table 8 indicate that the three principal components address different limitations of sparse-label soft sensing. They should not be interpreted as interchangeable accuracy-enhancement modules: the reconstruction branch changes how temporal information is learned, the monotonicity term changes how the regression response is regularized, and MC dropout changes how prediction reliability is characterized at inference time.

The self-supervised future state reconstruction branch allows the shared encoder to learn from the dense process trajectory without requiring additional slag assays. Predicting a future process state encourages the latent representation to retain information about temporal dependence and furnace state evolution between labeled assay times. This provides an auxiliary learning signal where direct quality supervision is sparse. Its role is representational rather than label generating, as the dense and overlapping process windows remain correlated and do not increase the number of independent slag tin targets.

The monotonicity constraint acts on the response surface rather than on temporal reconstruction. By penalizing positive prediction gradients with respect to the reduction potential index, it discourages locally implausible response directions that may otherwise be learned under limited supervision. Its effect should be judged jointly from point prediction metrics and physical behavior diagnostics. Lower PVR and MPG show greater agreement with the prescribed local direction, but do not establish that the proxy index is a complete causal or thermodynamic model.

MC dropout has a third role. The reconstruction and monotonicity terms shape the fitted model during training, whereas MC dropout samples the trained model during inference. The stochastic mean may slightly change the point estimate through model averaging, but the principal output added by this component is sample-dependent epistemic uncertainty. Its value is consequently assessed through calibration, interval width, and uncertainty–error association rather than through RMSE alone.

The small interaction contrasts show that the observed gains were approximately additive within the investigated experiment rather than being driven by a strong three-way interaction. This is consistent with a complementary division of labor: reconstruction supports temporal representation, monotonicity regularizes the response direction, and MC dropout reports model uncertainty. Integrating them allows one framework to address predictive accuracy, physically consistent behavior, and reliability reporting, while the strength and generality of these interactions still require validation on additional production periods and furnaces.

### 4.3. Physical Interpretation and Validity Range of the Monotonicity Constraint

The monotonicity constraint in PI-MTSS should be understood as a directional process prior rather than a complete first-principles description of the furnace. The reduction potential index retained in this study is defined as

(47) $$R_{t}={\mathrm{CO}}_{t}\cdot {\mathrm{Temp}}_{t},$$

where ${\mathrm{CO}}_{t}$ and ${\mathrm{Temp}}_{t}$ denote the mean CO concentration and mean furnace temperature within the input window, respectively. The two variables describe complementary aspects of the carbothermal reduction environment. CO is a major reducing species involved in tin oxide reduction and provides a sensor-available indication of the strength of the reducing atmosphere. Under otherwise comparable operating conditions, an increase in CO concentration generally indicates that a stronger reducing environment is available for removing oxygen from tin oxides; nevertheless, CO concentration alone cannot fully determine the furnace oxygen potential because it does not explicitly account for CO_2_, O_2_, gas mixing, or local reaction equilibrium.

Furnace temperature provides information about the thermal conditions under which reduction occurs. Temperature affects both the thermodynamic equilibrium and the kinetics of carbothermal reduction, including reaction rates, heat and mass transfer, and the development of the gas–slag–metal reaction environment. Within the stable operating region covered by the present dataset, sufficiently strong reducing conditions combined with an appropriate furnace temperature are generally associated with more complete tin reduction, and consequently with a lower residual tin content in the slag. The product form combines these two observable aspects into a simple macroscopic indicator; neither a reducing atmosphere nor a suitable thermal condition is considered in isolation.

This interpretation is deliberately local and conditional. The index does not represent a rigorously calculated oxygen potential, Gibbs free-energy change, or complete thermodynamic state of the furnace; instead, it expresses the expected response direction within the range of stable production conditions represented by the training data. Therefore, the monotonicity prior can be written as

(48) $$\frac{\partial {\hat{y}}_{t}}{\partial R_{t}}\leq 0,$$

meaning that an increase in the constructed reduction-potential indicator should not normally produce an increase in the predicted residual slag tin content. This type of directional regularization provides a practical compromise when complete governing equations and all thermodynamic state variables are unavailable [34,35].

To examine whether this behavior depends on the exact construction of the index, the product form was generalized in the sensitivity experiment as

(49) $$R_{t}^{\left(a,b\right)}={\left({\mathrm{CO}}_{t}\right)}^{a}{\left({\mathrm{Temp}}_{t}\right)}^{b},\;a>0,\;b>0,$$

where *a* and *b* control the relative sensitivity of the indicator to CO concentration and furnace temperature. These exponents are empirical sensitivity parameters rather than stoichiometric coefficients or thermodynamically identified constants. The neighboring exponent settings produced only small variations in RMSE and PVR, indicating that the regularization effect did not depend critically on one precisely specified pair of weights. Among the evaluated settings, the symmetric product with a=b=1 provided favorable average performance while retaining the most concise physical interpretation, and as such was preserved in the final model. However, this numerical stability only demonstrates robustness to moderate index perturbations within the investigated dataset, and should not be interpreted as rigorous thermodynamic validation of the selected formulation.

The assumed monotonicity also has explicit operational boundaries. It is introduced through a soft regularization term that is jointly optimized with the supervised regression and self-supervised reconstruction objectives rather than imposed as a hard law that must hold at every time point. Temporary deviations may occur during transient charging, adjustments of the blowing regime, abrupt changes in feed composition, incomplete bath mixing, sensor faults, or time-varying transport delays. Under these conditions, the measured CO concentration and temperature may not immediately represent the effective reaction conditions associated with a subsequent slag assay. The soft formulation allows the observed data to offset the prior when substantial contradictory evidence is present, thereby reducing the risk that an oversimplified physical assumption dominates model training.

Accordingly, PVR and MPG should be interpreted as model behavior diagnostics. A lower PVR indicates that fewer evaluated samples exhibit a positive prediction gradient with respect to the constructed index, whereas a lower MPG indicates that the remaining positive gradients have smaller magnitudes. Their reductions show that PI-MTSS responds more consistently with the prescribed inverse direction, but they do not prove that the index is causally correct, that the complete furnace state has been recovered, or that every physically plausible prediction has been obtained. A model may satisfy the monotonicity constraint while retaining biased point predictions, and an observed inverse association may also be influenced by unmeasured operating variables. Thus, PVR and MPG must be interpreted together with RMSE, MAE, $R^{2}$, temporal validation, and uncertainty calibration results.

Several alternative formulations could provide a more complete physical description when additional reliable measurements become available. First, the available CO and O_2_ measurements could be combined into an oxidation–reduction indicator in which CO contributes positively and O_2_ contributes negatively to the effective reduction tendency. Second, if reliable CO_2_ measurements become available, a CO/CO_2_ ratio or a temperature-dependent oxygen potential indicator could be constructed from gas-phase equilibrium relations. Third, slag composition, feed quantity and composition, blowing information, thermodynamic equilibrium calculations, and indicators of bath mixing could be incorporated to describe the reaction environment more comprehensively. Finally, a data-adaptive physical indicator could be learned using non-negative weights or derivative-sign constraints, so that its relative contributions are estimated from the data while their physically expected directions remain preserved.

These extensions would reduce reliance on the present two-variable proxy, but would also require additional sensor availability, reliable time alignment, and sufficient labeled operating coverage. Therefore, the current symmetric product should be regarded as a parsimonious and sensor-accessible prior that is suitable for the investigated production campaign. Its validity beyond the observed operating region must be assessed using longer production periods, different feed conditions, and independent furnace datasets before the assumed response direction can be generalized.

### 4.4. Reliability and Operational Interpretation of Uncertainty Estimation

Uncertainty reliability cannot be judged from PICP alone. PICP evaluates whether the empirical frequency of covered targets agrees with a nominal level, NMPIW evaluates the sharpness of those intervals relative to the training-period target range, and ACE summarizes coverage discrepancies across several nominal levels. A high PICP can be obtained with intervals that are too wide, while a small NMPIW is undesirable if it produces systematic undercoverage. Hence, these quantities must be interpreted jointly rather than ranked independently.

The intervals reported here are epistemic uncertainty intervals. They are derived solely from variation among stochastic MC dropout forward passes, and do not include any explicit residual noise, laboratory assay error, or process stochasticity terms. They mainly characterize sensitivity to uncertainty in the learned parameters and representations [24,25,26], and do not form a complete probabilistic description of aleatoric uncertainty in furnace operation.

Figure 4 and Table 11 provide complementary evidence. The proximity of empirical to nominal coverage addresses calibration, the normalized widths address sharpness, and the positive uncertainty–error association addresses whether the model assigns larger uncertainty to more difficult samples. The quartile analysis supports the latter interpretation because prediction errors increased with the uncertainty ranking. This ranking ability is distinct from calibration: a model can identify difficult samples while its intervals remain systematically too narrow or too wide, and a calibrated model can still rank individual errors poorly.

The estimates remain numerically coarse because the chronological test set contains only 40 assays. Within one model run, changing the coverage status of a single assay changes PICP by 2.5 percentage points. Averaging over five training seeds characterizes initialization and optimization variability, but all seeds are evaluated on the same assays and as such do not create additional independent test observations. The moving-block bootstrap partially reflects temporal sampling uncertainty without breaking the local chronological dependence, but cannot compensate for the limited duration and single-campaign scope of the dataset. Calibration should be reassessed after process drift, raw material changes, sensor replacement, equipment degradation, or model updating.

Operationally, a widened epistemic uncertainty interval should be treated as a request for additional scrutiny rather than as direct evidence of an abnormal furnace condition. A threshold selected from validation data could be used to prompt review of process measurements, request an additional slag assay, or temporarily increase laboratory sampling frequency. Persistent changes in uncertainty may also support drift detection and interval recalibration. These uses preserve the role of laboratory verification and operator knowledge instead of replacing them.

The present evidence is insufficient to support fully autonomous closed-loop control. Such use would require prospective evaluation of warning thresholds, costs related to false alarms and missed alarms, calibration stability under distribution shift, communication delays, and the consequences of incorrect control actions. In the current study, the uncertainty output should be regarded only as a reliability indicator for monitoring, review, and adaptive sampling.

### 4.5. Hyperparameter Robustness and Online Applicability

The sensitivity results should be interpreted according to the distinct functions of the investigated parameters. The input-window length *T* controls the temporal context presented to the encoder, $\lambda_{\mathrm{rec}}$ determines the strength of auxiliary dynamic representation learning, $\lambda_{\mathrm{phy}}$ controls the balance between data fitting and the monotonic prior, and the sampling number *N* controls the numerical stability and cost of MC dropout estimation. The relatively smooth changes around the selected settings support local robustness on the current validation period, but do not imply hyperparameter invariance across furnaces or production campaigns.

The window length pattern is consistent with a finite useful temporal context: a short window can truncate delayed responses between changes in feeding, reduction conditions, heat transfer, and the later slag assay, while a much longer window can include outdated states or combine observations from locally different regimes, thereby diluting recent information. Therefore, the selected window should be understood as an empirical context length for this dataset, not as an identified residence time or a causal delay.

The reconstruction coefficient controls competition between the auxiliary and primary objectives through the shared encoder. If its weight is too small, dense unlabeled trajectories provide little additional guidance; if it is too large, the network may prioritize reconstruction of process-variable fluctuations that are weakly related to slag tin content. The intermediate setting retains the regression task as the principal objective while allowing the auxiliary task to regularize temporal representation learning.

The physical loss coefficient creates a different trade-off: increasing it penalizes positive gradients more strongly, and consequently improves agreement with the prescribed inverse direction; however, excessive weighting can force the simplified proxy relationship onto transient or weakly represented conditions in which that prior is incomplete. This explains why physical consistency metrics can continue to improve after the point prediction error has begun to deteriorate. The selected coefficient is a data-dependent compromise, not a universal physical constant.

Increasing *N* reduces Monte Carlo sampling variability in the estimated mean and epistemic standard deviation, but does not reduce the underlying uncertainty of the trained model. The sensitivity and latency tables show diminishing changes in the uncertainty metrics while computational cost continues to grow with additional stochastic passes. This supports using the selected sampling number as a practical balance for the evaluated hardware and update frequency, rather than interpreting it as a universally optimal choice.

The deployment graph is lighter than the training graph because the reconstruction decoder and monotonicity gradient calculation are training-only operations. Online uncertainty estimation retains repeated stochastic passes, and is the principal model-side latency source. The historical window represents a rolling context buffer, so its duration is not the prediction delay; after initialization, the buffer can be updated whenever a new process record arrives. The measured latency is well below the minute-scale data-update interval on the evaluated workstation, supporting model-side feasibility for operator decision support. End-to-end industrial feasibility remains unverified because the timing experiment excluded process signal acquisition, DCS communication, databases, data quality checks, visualization, network behavior, operator interaction, maintenance procedures, and actuator execution. Prospective industrial validation remains necessary to evaluate long-term computational stability, communication failures, process drift, warning thresholds, and the operational consequences of model errors.

## 5. Conclusions

This study develops PI-MTSS for uncertainty-aware soft sensing of slag tin content in top-blown tin smelting under sparse laboratory supervision. The framework employs a shared TCN-based temporal encoder to extract furnace dynamics from high-frequency process measurements. A self-supervised future state reconstruction branch exploits unlabeled process trajectories, while a supervised regression branch estimates slag tin content at assay times. In addition, a soft monotonicity constraint based on a simplified CO–temperature reduction potential indicator improves consistency with the expected process response direction, and MC dropout provides sample-dependent epistemic uncertainty estimates.

Experiments were conducted using 43,200 one-minute process observations and 240 laboratory labels, including 40 chronologically held-out test labels. PI-MTSS achieved an RMSE of 0.198, an MAE of 0.145, and an $R^{2}$ of 0.889, outperforming the evaluated baseline models. The complete model reduced the physical violation rate to 3.5% and the mean positive gradient to 0.004, confirming that the reconstruction task improved sparse-label learning and that the monotonicity constraint reduced responses inconsistent with the prescribed prior. At the 95% nominal level, the epistemic uncertainty intervals achieved a seed-averaged PICP of 96.5%, an NMPIW of 0.245, and an ACE of 2.08 percentage points, indicating slightly conservative coverage with a reasonable calibration–width balance. The measured inference time was also below the one-minute data update interval on the evaluated hardware, supporting minute-scale monitoring and operator decision support.

The present evidence is limited to one continuous month from a single furnace, and the small test set introduces considerable uncertainty into performance and interval calibration estimates. Moreover, the simplified reduction potential indicator does not fully represent oxygen potential, slag chemistry, feed variations, or bath mixing, while MC dropout mainly captures epistemic rather than total process uncertainty. Future work will: conduct external validation across multiple campaigns, furnaces, and raw material batches; introduce drift detection, domain adaptation, periodic recalibration, and uncertainty-triggered laboratory sampling; and develop richer thermodynamic indicators while comparing MC dropout with deep ensembles and time-aware conformal prediction. Further industrial validation is required before the method can be extended from decision support to autonomous closed-loop control.

## Figures and Tables

**Table 1:** Confidentiality-preserving overview of the industrial dataset.

Data Category	Variables	Acquisition Source	Temporal Characteristic	Modeling Role
Process measurements	Temperature, CO, O_2_, and SO_2_	Distributed control system	High-frequency synchronous records	Model inputs and reconstruction targets
Quality measurement	Slag tin content	Offline laboratory assay	Sparse scheduled observations	Supervised regression target

**Table 2:** Implementation settings used for PI-MTSS.

Component	Hyperparameter	Final Setting
Input construction	Window length	6 h (360 one-minute steps)
TCN encoder	Blocks, channel widths	4, [64,128,128,128]
TCN encoder	Kernel size, dilation factors	3, [1,2,4,8]
Temporal aggregation	Operation, latent dimension	Adaptive average pooling, 128
Future-state decoder	Layer dimensions	128→64→4
Regression head	Layer dimensions	129→64→32→1
Regularization	Dropout probability	0.20
Optimization	Optimizer, learning rate	AdamW, $1\times 10^{-3}$
Optimization	Weight decay, mini-batch size	$1\times 10^{-4}$, 32 (8 labeled + 24 unlabeled)
Optimization	Maximum epochs, early-stopping patience	300, 30 epochs
Multi-task learning	$\left(\lambda_{\mathrm{rec}},\lambda_{\mathrm{phy}}\right)$	(0.1,0.5)
Repeated training	Random seeds	{0,1,2,3,4}
Uncertainty inference	MC-Dropout passes	50

**Table 3:** Point prediction performance of PI-MTSS and the baseline models on the chronologically held-out testing set. Neural model results are reported as mean ± standard deviation over five random seeds. The downward arrow (↓) denotes that a lower value is preferable, whereas the upward arrow (↑) denotes that a higher value is preferable. The arrows in the subsequent table have the same meaning.

Model	RMSE ↓	MAE ↓	$R^{2}$↑
SVR	0.312	0.245	0.724
LSTM	0.248±0.006	0.186±0.005	0.826±0.008
Vanilla TCN	0.215±0.005	0.158±0.004	0.869±0.006
PI-MTSS	0.198±0.004	0.145±0.003	0.889±0.005

**Table 4:** Statistical comparison of PI-MTSS with the baseline models. A positive $\Delta_{\mathrm{RMSE}}$ favors PI-MTSS. CI denotes the temporal block-bootstrap confidence interval and $p_{\mathrm{Holm}}$ denotes the Holm-adjusted paired block-permutation *p*-value.

Reference Model	$\Delta_{\mathrm{RMSE}}$	Relative Reduction (%)	95% CI	$p_{\mathrm{Holm}}$	Decision at α=0.05
SVR	0.114	36.54	[0.076,0.151]	0.006	Significant
LSTM	0.050	20.16	[0.018,0.080]	0.024	Significant
Vanilla TCN	0.017	7.91	[−0.002,0.036]	0.094	Not significant

**Table 5:** Chronological partitions used for rolling-origin temporal validation.

Window	Training Period	Validation Period	Testing Period	Labeled Assays (Training/Validation/Testing)
W1	P1–P2	P3	P4	80/40/40
W2	P1–P3	P4	P5	120/40/40
W3	P1–P4	P5	P6	160/40/40

**Table 6:** Rolling-window temporal validation results. The results in each window are seed-averaged values, whereas the summary rows report mean ± standard deviation across the three testing periods.

Window (Test Period)	Model	RMSE ↓	MAE ↓	$R^{2}$↑
W1 (P4)	SVR	0.326	0.258	0.701
W1 (P4)	LSTM	0.263	0.198	0.804
W1 (P4)	Vanilla TCN	0.229	0.170	0.848
W1 (P4)	PI-MTSS	**0.210**	**0.155**	**0.872**
W2 (P5)	SVR	0.298	0.232	0.742
W2 (P5)	LSTM	0.241	0.181	0.836
W2 (P5)	Vanilla TCN	0.207	0.152	0.879
W2 (P5)	PI-MTSS	**0.190**	**0.139**	**0.900**
W3 (P6)	SVR	0.312	0.245	0.724
W3 (P6)	LSTM	0.248	0.186	0.826
W3 (P6)	Vanilla TCN	0.215	0.158	0.869
W3 (P6)	PI-MTSS	**0.198**	**0.145**	**0.889**
Across-window	SVR	0.312±0.014	0.245±0.013	0.722±0.021
Across-window	LSTM	0.251±0.011	0.188±0.009	0.822±0.016
Across-window	Vanilla TCN	0.217±0.011	0.160±0.009	0.865±0.016
Across-window	PI-MTSS	0.199±0.010	0.146±0.008	0.887±0.014

**Table 7:** Component interaction quantities calculated from the mean results in Table 8. Positive pairwise values indicate a joint improvement beyond the sum of the individual component effects. The three-factor contrast measures the change in the A–B interaction with MC dropout inference enabled.

Metric	$I_{\mathrm{AB},0}$	$I_{\mathrm{AB},1}$	$I_{\mathrm{ABC}}$
RMSE reduction	0.002	0.002	0.000
MAE reduction	0.001	0.000	−0.001
$R^{2}$ increase	0.005	0.004	−0.001

**Table 8:** Full component ablation results on the chronologically held-out testing set. A, B, and C denote self-supervised future state reconstruction, physics-informed monotonicity regularization, and MC dropout inference, respectively. A value of 1 indicates that the component was enabled. All results are reported as mean ± standard deviation over five random seeds.

Configuration	Components			Point Prediction			Uncertainty Quantification		
	A	B	C	RMSE ↓	MAE ↓	$R^{2}$ ↑	PICP_90_(%) ↑	NMPIW_90_ ↓	ACE ↓
V0: Base TCN	0	0	0	0.215±0.005	0.158±0.004	0.869±0.006	–	–	–
V1: A	1	0	0	0.206±0.005	0.151±0.004	0.878±0.006	–	–	–
V2: B	0	1	0	0.211±0.005	0.155±0.004	0.872±0.006	–	–	–
V3: C	0	0	1	0.213±0.005	0.157±0.004	0.871±0.006	82.0±2.2	0.252±0.008	0.071±0.006
V4: A+B	1	1	0	0.200±0.004	0.147±0.003	0.886±0.005	–	–	–
V5: A+C	1	0	1	0.204±0.004	0.149±0.004	0.881±0.005	87.5±1.8	0.229±0.007	0.039±0.005
V6: B+C	0	1	1	0.209±0.005	0.153±0.004	0.875±0.006	85.0±2.0	0.238±0.007	0.052±0.006
V7: A+B+C (PI-MTSS)	1	1	1	0.198±0.004	0.145±0.003	0.889±0.005	90.5±1.4	0.211±0.006	0.021±0.004

**Table 9:** Physical consistency results on the chronologically held-out testing set. A, B, and C denote self-supervised future state reconstruction, physics-informed monotonicity regularization, and MC dropout inference, respectively. All values are reported as mean ± standard deviation over five random seeds.

Configuration	A	B	C	PVR (%) ↓	MPG ↓	Median *g*	$Q_{0.95}\left(g\right)$
V0: Base TCN	0	0	0	42.5±4.7	0.083±0.011	−0.041±0.019	0.331±0.043
V1: A	1	0	0	35.5±4.1	0.061±0.009	−0.089±0.017	0.276±0.038
V2: B	0	1	0	8.0±2.7	0.010±0.003	−0.214±0.015	0.024±0.009
V3: C	0	0	1	40.5±4.2	0.078±0.010	−0.049±0.018	0.315±0.041
V4: A+B	1	1	0	5.5±2.0	0.006±0.002	−0.239±0.014	0.007±0.006
V5: A+C	1	0	1	32.0±3.8	0.055±0.008	−0.102±0.016	0.251±0.035
V6: B+C	0	1	1	7.0±2.2	0.008±0.002	−0.222±0.014	0.018±0.008
V7: A+B+C (PI-MTSS)	1	1	1	3.4±1.4	0.004±0.001	−0.257±0.013	−0.009±0.005

**Table 10:** Measured and predicted slag tin contents under different reduction potential levels. Values of $\tilde{R}$ and slag tin content are reported as mean ± standard deviation across the samples in each group. Group RMSE values are seed-averaged results.

Reduction-Potential Level	*n*	Mean $\tilde{R}$	Measured Sn (wt.%)	Base TCN Prediction (wt.%)	PI-MTSS Prediction (wt.%)	Base TCN RMSE	PI-MTSS RMSE
Low	13	−0.91±0.31	2.35±0.45	2.16±0.40	2.30±0.42	0.236	**0.214**
Medium	14	−0.04±0.23	1.82±0.50	1.87±0.42	1.81±0.46	0.208	**0.190**
High	13	0.94±0.34	1.31±0.35	1.50±0.33	1.35±0.33	0.199	**0.189**

**Table 11:** Uncertainty calibration and reliability results on the chronologically held-out testing set. A, B, and C denote self-supervised future state reconstruction, physics-informed monotonicity regularization, and MC dropout inference, respectively. PICP values should be interpreted according to their deviation from the corresponding nominal coverage rather than as metrics for which an arbitrarily larger value is always preferable.

Configuration	PICP_90_ (%)	NMPIW_90_	PICP_95_ (%)	NMPIW_95_	ACE ↓	Spearman $\rho_{s}$↑
V3: C	82.0±2.2	0.252±0.008	84.3±2.2	0.325±0.010	0.071±0.006	0.438±0.055
V5: A+C	87.5±1.8	0.229±0.007	92.4±1.8	0.278±0.008	0.039±0.005	0.612±0.047
V6: B+C	85.0±2.0	0.238±0.007	88.5±2.0	0.295±0.009	0.052±0.006	0.548±0.051
V7: A+B+C (PI-MTSS)	90.5±1.4	0.211±0.006	96.5±1.4	0.245±0.007	0.021±0.004	0.691±0.042

**Table 12:** Sensitivity of PI-MTSS to the input window length, loss coefficients, and reduction potential formulation on the chronological validation period. Only one factor was varied at a time. Bold values indicate the default configuration. PVR and MPG evaluate physical consistency rather than point prediction accuracy.

Candidate Value	RMSE ↓	MAE ↓	$R^{2}$↑	PVR (%) ↓	MPG ↓
*Input window length T*					
2h	0.216±0.008	0.159±0.006	0.867±0.009	8.5±2.2	0.014±0.003
4h	0.206±0.006	0.151±0.005	0.879±0.007	5.5±2.0	0.008±0.002
6h	0.201±0.005	0.147±0.004	0.885±0.006	4.0±1.4	0.005±0.001
8h	0.204±0.006	0.149±0.004	0.882±0.007	4.5±1.4	0.006±0.002
10h	0.210±0.007	0.154±0.005	0.874±0.008	5.5±2.0	0.008±0.002
*Reconstruction loss coefficient $\lambda_{\mathrm{rec}}$*					
0	0.209±0.006	0.153±0.005	0.876±0.007	6.5±2.2	0.009±0.002
0.05	0.204±0.006	0.150±0.004	0.882±0.007	5.0±1.8	0.007±0.002
0.1	0.201±0.005	0.147±0.004	0.885±0.006	4.0±1.4	0.005±0.001
0.2	0.203±0.006	0.149±0.004	0.883±0.007	4.5±1.4	0.006±0.002
0.5	0.211±0.007	0.155±0.005	0.873±0.008	5.5±2.0	0.008±0.002
*Monotonicity loss coefficient $\lambda_{\mathrm{phy}}$*					
0	0.207±0.006	0.152±0.005	0.878±0.007	31.5±4.1	0.056±0.008
0.1	0.204±0.006	0.150±0.004	0.882±0.007	13.0±2.7	0.020±0.004
0.25	0.202±0.005	0.148±0.004	0.884±0.006	7.5±2.0	0.010±0.003
0.5	0.201±0.005	0.147±0.004	0.885±0.006	4.0±1.4	0.005±0.001
1.0	0.204±0.006	0.150±0.004	0.882±0.007	2.5±1.1	0.003±0.001
2.0	0.212±0.008	0.157±0.006	0.872±0.009	1.0±0.9	0.001±0.001
*Reduction potential exponents (a,b)*					
(0.8,1.2)	0.204±0.006	0.150±0.004	0.882±0.007	5.5±2.0	0.007±0.002
(0.9,1.1)	0.202±0.005	0.148±0.004	0.884±0.006	4.5±1.4	0.006±0.002
(1.0,1.0)	0.201±0.005	0.147±0.004	0.885±0.006	4.0±1.4	0.005±0.001
(1.1,0.9)	0.202±0.005	0.148±0.004	0.884±0.006	4.5±1.4	0.006±0.002
(1.2,0.8)	0.205±0.006	0.151±0.005	0.880±0.007	6.0±2.2	0.008±0.002

**Table 13:** Sensitivity of uncertainty estimation to the number of MC dropout stochastic forward passes on the chronological validation period. The relative sampling cost is the number of forward passes normalized by the selected setting N=50, and does not represent measured wall-clock time. PICP should be interpreted according to its deviation from the nominal 95% coverage.

*N*	PICP_95_ (%)	NMPIW_95_	ACE ↓	Spearman $\rho_{s}$ ↑	Relative Sampling Cost
10	89.5±2.7	0.226±0.011	0.051±0.007	0.604±0.058	0.20
20	92.5±2.2	0.236±0.009	0.036±0.006	0.649±0.052	0.40
30	94.5±1.8	0.242±0.008	0.028±0.005	0.674±0.047	0.60
50	95.5±1.4	0.246±0.007	0.022±0.004	0.687±0.043	1.00
100	95.5±1.4	0.247±0.007	0.021±0.004	0.692±0.042	2.00

**Table 14:** Computational complexity of PI-MTSS for a single 360-step input window. The deployed graph excludes the training-only reconstruction decoder and monotonicity loss calculation.

Computational Item	Complete Training Graph	Online Point Prediction	Online Uncertainty Estimation (N=50)
Active parameters (million)	0.318	0.304	0.304
Serialized FP32 model size (MB)	1.31	1.25	1.25
Forward-pass complexity (GFLOPs/window)	0.041	0.038	1.900
Number of forward passes per update	1	1	50
Reconstruction decoder	Included	Removed	Removed
Monotonicity gradient calculation	Included	Disabled	Disabled

**Table 15:** Online inference latency of PI-MTSS on the NVIDIA GeForce RTX 4050 GPU. The update-budget occupation is calculated relative to the one-minute sampling interval for process-data.

Inference Mode	Sampling Number *N*	Mean Latency (ms)	95th-Percentile Latency (ms)	Relative Cost	60-s Update-Budget Occupation (%)
Point prediction	1	1.3±0.1	1.5	0.02	0.002
MC dropout	10	12.0±0.4	12.7	0.21	0.020
MC dropout	20	23.6±0.6	24.7	0.40	0.039
MC dropout	30	35.2±0.8	36.6	0.60	0.059
MC dropout	50	58.4±1.2	60.5	1.00	0.097
MC dropout	100	116.2±2.1	119.8	1.99	0.194

## Data Availability

The data supporting the findings of this study are not publicly available due to industrial confidentiality and proprietary restrictions. The relevant experimental results and processed analyses are presented in the manuscript.

## Abbreviations

The following abbreviations are used in this manuscript:

DCS	Distributed Control System
LSTM	Long Short-Term Memory
TCN	Temporal Convolutional Network
OOD	Out-of-Distribution
MC Dropout	Monte Carlo Dropout
PI-MTSS	Physics-Informed Multi-Task Soft Sensor
SVR	Support Vector Regression
RMSE	Root Mean Square Error
MAE	Mean Absolute Error
$R^{2}$	Coefficient of determination
PICP	Prediction Interval Coverage Probability
NMPIW	Normalized Mean Prediction Interval Width
ACE	Average Calibration Error
PVR	Physical Violation Rate
MPG	Mean Positive Gradient Magnitude
